# Planned mode of birth after previous cesarean section and risk of undergoing pelvic floor surgery: A Scottish population-based record linkage cohort study

**DOI:** 10.1371/journal.pmed.1004119

**Published:** 2022-11-22

**Authors:** Kathryn E. Fitzpatrick, Mohamed Abdel-Fattah, Joris Hemelaar, Jennifer J. Kurinczuk, Maria A. Quigley

**Affiliations:** 1 National Perinatal Epidemiology Unit, Nuffield Department of Population Health, University of Oxford, Oxford, United Kingdom; 2 The Aberdeen Centre for Women’s Health Research, University of Aberdeen, Aberdeen, United Kingdom; 3 Department of Obstetrics, Women’s Centre, John Radcliffe Hospital, Oxford University Hospitals NHS Foundation Trust, Oxford, United Kingdom

## Abstract

**Background:**

The global rise in cesarean sections has led to increasing numbers of pregnant women with a history of previous cesarean section. Policy in many high-income settings supports offering these women a choice between planned elective repeat cesarean section (ERCS) or planned vaginal birth after previous cesarean (VBAC), in the absence of contraindications to VBAC. Despite the potential for this choice to affect women’s subsequent risk of experiencing pelvic floor disorders, evidence on the associated effects to fully counsel women is lacking. This study investigated the association between planned mode of birth after previous cesarean section and the woman’s subsequent risk of undergoing pelvic floor surgery.

**Methods and findings:**

A population-based cohort study of 47,414 singleton term births in Scotland between 1983 to 1996 to women with 1 or more previous cesarean sections was conducted using linked Scottish national routine datasets. Cox regression was used to investigate the association between planned as well as actual mode of birth and women’s subsequent risk of having any pelvic floor surgery and specific types of pelvic floor surgery adjusted for sociodemographic, maternal medical, and obstetric-related factors. Over a median of 22.1 years of follow-up, 1,159 (2.44%) of the study population had pelvic floor surgery. The crude incidence rate of any pelvic floor surgery per 1,000 person-years was 1.35, 95% confidence interval (CI) 1.27 to 1.43 in the overall study population, 1.75, 95% CI 1.64 to 1.86 in the planned VBAC group and 0.66, 95% CI 0.57 to 0.75 in the ERCS group. Planned VBAC compared to ERCS was associated with a greater than 2-fold increased risk of the woman undergoing any pelvic floor surgery (adjusted hazard ratio [aHR] 2.38, 95% CI 2.03 to 2.80, *p* < 0.001) and a 2- to 3-fold increased risk of the woman having surgery for pelvic organ prolapse or urinary incontinence (aHR 3.17, 95% CI 2.47 to 4.09, *p* < 0.001 and aHR 2.26, 95% CI 1.79 to 2.84, *p* < 0.001, respectively). Analysis by actual mode of birth showed these increased risks were only apparent in the women who actually had a VBAC, with the women who needed an in-labor non-elective repeat cesarean section having a comparable risk of pelvic floor surgery to those who had an ERCS. The main limitation of this study is the potential for misclassification bias.

**Conclusions:**

This study suggests that among women with previous cesarean section giving birth to a singleton at term, planned VBAC compared to ERCS is associated with an increased risk of the woman subsequently undergoing pelvic floor surgery including surgery for pelvic organ prolapse and urinary incontinence. However, these risks appear to be only apparent in women who actually give birth vaginally as planned, highlighting the role of vaginal birth rather than labor in pelvic floor dysfunction requiring surgery. The findings provide useful additional information to counsel women with previous cesarean section about the risks and benefits associated with their future birth choices.

## Abstract 2

In a Scottish population-based record linkage cohort study, Dr Kathryn E. Fitzpatrick and colleagues, investigate planned mode of birth and the risk of subsequent pelvic floor surgery following previous cesarean section.

## Author summary

**Why was this study done?:**

- The global rise in cesarean section rates has led to increasing numbers of pregnant women with a history of previous cesarean section.
- Pregnant women with a history of previous cesarean section may be given a choice between planned elective repeat cesarean section (ERCS) or planned vaginal birth providing they do not have medical reasons why a vaginal birth is not recommended.
- While clinical guidelines recommend that women should be counseled on the associated risks and benefits to help them make an informed decision about this choice, there is a lack of evidence about the effect of this choice on long-term outcomes including women’s subsequent risk of experiencing pelvic floor disorders including pelvic organ prolapse, urinary incontinence, rectal prolapse, and fecal incontinence.

**What did the researchers do and find?:**

- We conducted a cohort study of 47,414 singleton term births in Scotland to women with 1 or more previous cesarean sections to determine the association between planned as well as actual mode of birth after previous cesarean section and the woman’s subsequent risk of undergoing pelvic floor surgery.
- We found that planned vaginal birth compared to planned ERCS is associated with a greater than 2-fold increased risk of the woman undergoing any pelvic floor surgery and a 2- to 3-fold increased risk of the woman having surgery for pelvic organ prolapse or urinary incontinence.
- However, the increased risks seen in the planned vaginal birth group were only apparent in the women who actually gave birth vaginally as planned. Women who needed a non-elective repeat cesarean section during labor have a similar risk of pelvic floor surgery to those who had a planned ERCS.

**What do these findings mean?:**

- Our findings provide additional information to counsel women with previous cesarean section about the risks and benefits associated with their future birth choices.

## Introduction

Cesarean section rates have risen sharply in many parts of the world, including the United Kingdom where over 30% of all births now occur by cesarean section [1–3]. An increasing number of women are therefore embarking on a subsequent pregnancy with a cesarean scar. Current policy consensus in high-income settings supports offering these women a choice between planning another cesarean section, known as an elective repeat cesarean section (ERCS) or planning a vaginal birth, commonly known as a planned vaginal birth after previous cesarean (VBAC), providing they do not have contraindications to VBAC [4–8]. Clinical guidelines [4–8] also advocate counseling women on the risks and benefits of their options to facilitate informed decision-making. The existing evidence suggests that while planned VBAC compared to ERCS is associated with an increased risk of various serious birth-related complications for both the woman and her baby, the absolute risk of birth-related complications is small for either birth approach [9–12]. Also, while repeat cesarean section has been linked to an increased risk of maternal complications in subsequent pregnancies such as morbidly adherent placenta [10], we have recently found little evidence to support an association between planned mode of birth after previous cesarean section and special education needs in childhood, a marker of neurodevelopmental adversity in the child [13]. However, there remains a lack of evidence about the effect of this choice on other important long-term outcomes including women’s subsequent risk of experiencing pelvic floor disorders [14].

Pelvic floor disorders, including pelvic organ prolapse, urinary incontinence, and fecal incontinence, affect many women globally, particularly older parous women [15–18]. These disorders can have a substantial adverse impact on women’s well-being and quality of life [19,20]. While conservative management is generally the first line of treatment, the lifetime risk of undergoing surgery for pelvic floor disorders has been estimated at around 1 in 8 among parous women in the UK [21] and as high as 1 in 5 among women in the United States [22]. There is conflicting evidence and ongoing controversy as to whether planned/pre-labor cesarean section confers protection against pelvic floor disorders [23–27]. This may partly reflect limitations with the existing studies, including small sample sizes, short periods of follow-up, not accounting for preexisting pelvic floor dysfunction, and/or a focus on actual mode of birth without differentiating between pre-labor and in-labor cesarean section. Also, to the best of our knowledge, no studies have specifically examined the effect of planned mode of birth after previous cesarean on women’s subsequent risk of experiencing pelvic floor disorders [14]. However, several recent studies [28–30] have reported that VBAC compared to vaginal birth in primiparous women is associated with an increased risk of obstetric anal sphincter injury, a recognized risk factor for certain subsequent pelvic floor disorders such as fecal incontinence [31–33]. There is a need for information about women’s subsequent risk of experiencing pelvic floor disorders according to planned mode of birth after previous cesarean section to appropriately counsel women with previous cesarean section and facilitate informed decision-making about their birth choices. The aim of this study was to investigate the association between planned mode of birth after previous cesarean section and the woman’s subsequent risk of undergoing pelvic floor surgery as an indication of their risk of having a pelvic floor disorder sufficiently severe as to require surgical intervention, among women giving birth to a singleton at term and considered clinically eligible to plan VBAC.

## Methods

The study is reported as per the Reporting of studies Conducted using Observational Routinely collected Data (RECORD) guideline (S1 File).

### Study design and data sources

Scotland has a long history of collecting high-quality routine population-based health-related information, with linkable data readily available from the early 1980s. This data not only captures a wealth of information about women’s reproductive history but also provides a cost-effective and timely way to study outcomes such as subsequent pelvic floor surgery that may not take place for many years [21]. A population-based cohort study was conducted by linking a total of 5 Scottish national datasets (Table A in S2 File) using exact matching of the Community Health Index number, a unique 10-digit person identifier used in Scotland. The data sources, codes, and database fields that were used are shown in Table B in S2 File.

### Study population

All women with 1 or more previous cesarean sections who gave birth to a term (37 to 41 completed weeks gestation) singleton in Scotland, UK, between 1 January 1983 and 31 December 1996 were identified. This time period was chosen by considering the availability of the data used at the time it was requested and to allow follow-up for at least 20 years, recognizing that the outcomes of interest may not take place for many years [21]. Births to women not considered clinically eligible to plan a VBAC based on UK guidelines [4,5] were excluded (Table B in S2 File) along with births to women who had an antepartum stillbirth as vaginal birth is usually recommended in this situation [34]. To identify incident events of pelvic floor surgery, births to women with any evidence of pelvic floor surgery before they gave birth were excluded. Further exclusions included births with missing information about mode of birth and gestational age, births by non-elective cesarean section missing information about duration of labor, and births in which the number of previous cesarean sections was greater than a woman’s recorded parity. Each woman was followed up from the date of their first eligible birth until the outcome of interest, date of emigration, death, or 31 December 2016 (the most recent data available at the time it was requested), whichever came first. If a woman had more than 1 birth during the follow-up period, the time to event was restarted after each successive birth, with the exposures and covariates treated as time-varying variables that were reassessed at each pregnancy/birth (Fig A and Table B in S2 File).

### Exposure variables

The main exposure of interest was planned mode of birth after previous cesarean, with planned VBAC compared to ERCS. Planned VBAC was defined as birth vaginally or by non-elective cesarean section with a duration of labor of ≥1 hour, with duration of labor defined by ISD Scotland as “the length of time the state of labor lasts from its onset to the delivery of the placenta, expressed as the number of completed hours.” ERCS was defined as birth by elective cesarean section, defined by ISD Scotland as a “cesarean performed during the day with both the patient and staff fully prepared.”

Recognizing the potential of labor induction to increase the risk of pelvic floor disorders [35,36], analysis was also conducted according to whether planned VBAC was attempted with or without labor induction compared to ERCS. Analysis was also performed according to actual mode of birth after previous cesarean, defined as follows: women who were recorded as having a vaginal birth were classified as having a VBAC; women who were recorded as having a non-elective cesarean section with a duration of labor of ≥1 hour were classified as having an in-labor non-elective repeat cesarean section; and women who were recorded as having an elective cesarean section were classified as having an ERCS.

### Outcomes

The primary outcome was time to first pelvic floor surgery, including surgical treatment for any of the following: pelvic organ prolapse, urinary incontinence, rectal prolapse, or fecal incontinence. Secondary outcomes were time to first surgery for specific types of pelvic floor disorders, analyzing pelvic organ prolapse, urinary incontinence, and rectal prolapse or fecal incontinence as 3 separate outcomes.

### Statistical analysis

All analyses were prespecified as described in the methods, with the exception of calculating population attributable fractions (PAFs) and a post-hoc sensitivity analysis defining planned VBAC using slightly different criteria as described below. We did not publish or preregister an analysis plan, but a summary of the proposed study exposures, outcomes, and statistical methods was included as part of the application to the Public Benefit and Privacy Panel for Health and Social Care Scotland to obtain the data (Text A in S2 File) and as part of the funding application to the National Institute for Health and Care Research (NIHR). Kaplan–Meier failure curves were plotted for the primary outcome according to planned mode of birth after previous cesarean section. To examine the association between the exposures and each outcome, Cox proportional hazards models were used to estimate hazard ratios (HRs) with accompanying 95% confidence intervals (CIs) and Wald test *p*-values. To account for temporal changes, models were first adjusted for year of birth. Models were then adjusted in a hierarchical fashion for covariates determined a priori based on preexisting hypotheses or evidence of what factors are thought to potentially confound the investigated associations [21,37]: model A was adjusted for sociodemographic factors; model B was additionally adjusted for a priori maternal medical and obstetric-related factors (Table B in S2 File). Whether the woman had any prior vaginal births and parity were investigated a priori as potential effect modifiers of the relationship between the primary exposure and outcomes by the addition of interaction terms to the fully adjusted models, with Wald tests of the interaction terms used to calculate *p*-values for the interaction. Fractional polynomials were used to investigate whether continuous variables showed evidence of departure from linearity. Multiple imputation using an extension to the chained equations approach [38] was used to impute partially observed covariates when there was evidence from the complete case analysis of effect modification or nonlinear covariate effects. Otherwise, multiple imputation using the normal chained equations method was used including the event indicator and Nelson–Aalen estimator of the cumulative hazard [39] in the imputation models and all covariates, and performing 20 imputations. The proportional hazards assumption was verified visually using log–log plots. Robust standard errors were used to account for the lack of independence in the data of women who had more than 1 eligible birth in the study period. PAFs associated with planned VBAC were estimated as: (proportion of women exposed among those with the outcome of interest) x (aHR-1/aHR), where aHR is the fully adjusted hazard ratio from the Cox proportional hazards model. The Bonferroni inequality method [40] was used to calculate approximate 95% CIs for the PAFs. All *p*-values were 2-sided with the significance level set at <0.05.

Several prespecified sensitivity analyses were conducted including a complete case analysis for each outcome investigated, calculating E-values to assess the potential effect of unmeasured confounding [41], conducting analyses in the subgroup of women who had all their previous births captured in the dataset allowing for the adjustment of additional covariates (Table B in S2 File), and analyzing the association between the exposures and the primary outcome in the subgroup of women who gave birth between 1 January 1983 and 31 December 1986 to allow follow-up for at least 30 rather than 20 years. Following reviewer feedback, an additional sensitivity analysis was conducted, defining planned VBAC as birth vaginally or by non-elective cesarean section with a duration of labor of >4 hours (rather than ≥1 hour). All analyses were conducted in StataMP version 16.

## Results

**Fig 1:**
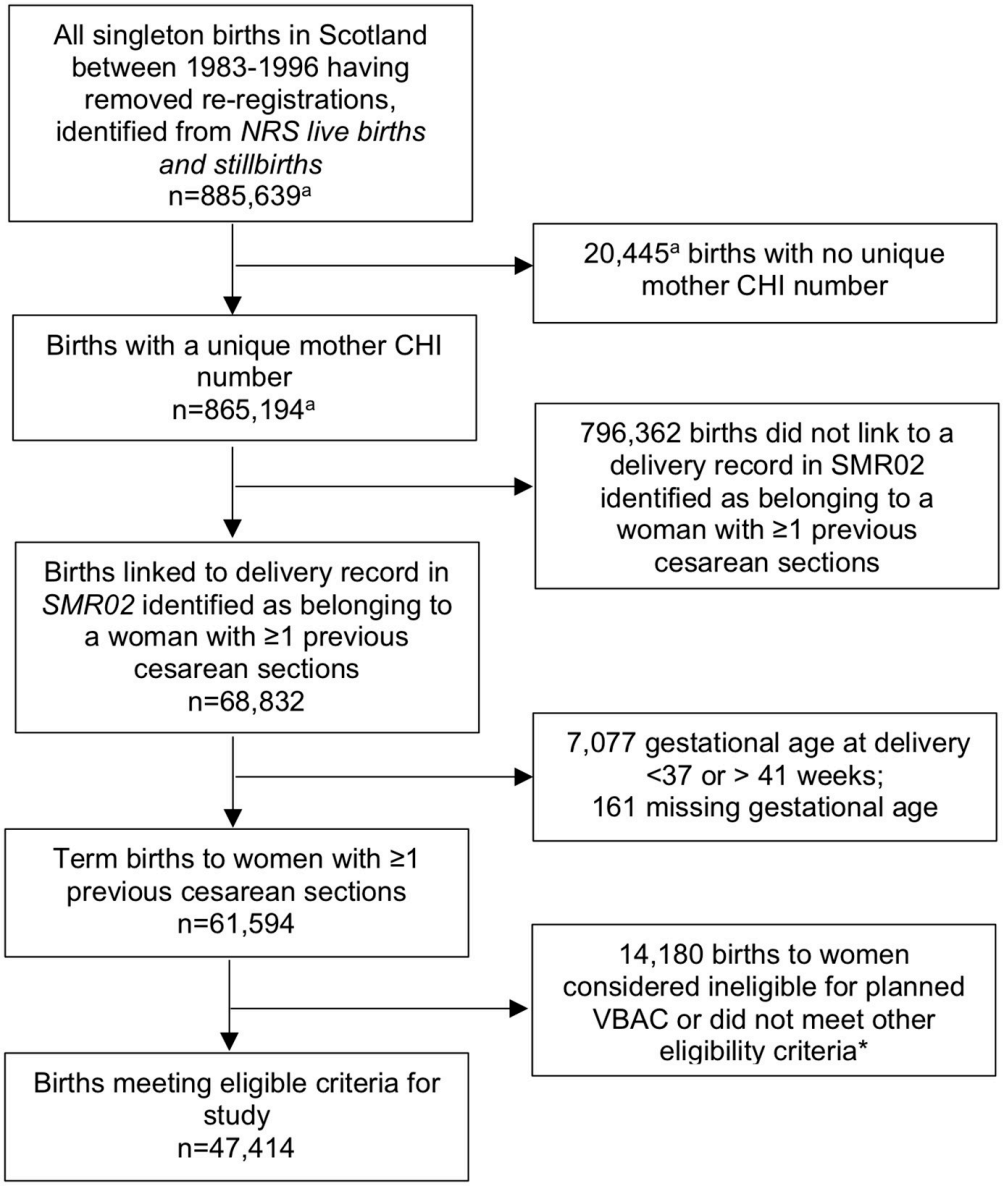
Flow diagram of cohort selection. *Ineligible for planned VBAC or did not meet other eligibility criteria for study due to 1 or more of the following: non-cephalic presentation at delivery (*n* = 4,556); placenta praevia (*n* = 546); abdominal pregnancy (*n* = 1); known or suspected disproportion of maternal and/or fetal origin (*n* = 6,593); tumor of corpus uteri (*n* = 97); birth by pre-labor non-elective cesarean section (*n* = 2,632); antepartum stillbirth (*n* = 0); stillbirth missing time of death in relation to delivery (*n* = 147); pelvic floor surgery before birth (*n* = 28); missing information on mode of delivery (*n* = 24); delivered by non-elective cesarean section missing information about duration of labor (*n* = 927); number of previous cesarean sections greater than parity (*n* = 15). Reasons not mutually exclusive. ^a^Numbers provided by ISD Scotland, now part of Public Health Scotland. CHI, community health index; ISD, Information Services Division; NRS, National Records of Scotland; SMR02, Scottish Morbidity Record Maternity Inpatient and Day Case dataset; VBAC, vaginal birth after previous cesarean.

A total of 47,414 singleton term births to women with 1 or more previous cesarean sections met the study eligibility criteria (Fig 1), 66.8% (31,672) of whom had a planned VBAC and 33.2% (15,742) had an ERCS. Over the study period, a small increase in the ERCS rate was seen from 34.1% in 1983 to 37.0% in 1996. Women who planned a VBAC were more likely than those who had an ERCS to be younger and were more likely to have had 1 or more prior vaginal birth (median number of prior vaginal births in women with any prior vaginal births: 1, interquartile range (IQR) 1–2 in planned VBAC group and 1, IQR 1–1 in ERCS), a shorter interpregnancy interval, and a baby with a birth weight of 4,000 g or more (Table 1). They were less likely than women who had an ERCS to be married, to have a parity of 2 or more, and to have diabetes.

**Table 1:** Characteristics of the study cohort by planned mode of birth after previous cesarean section.

	ERCS n(%)^a^ unless otherwise stated (*n* = 15,742)	Planned VBAC n(%)^a^ unless otherwise stated (*n* = 31,672)
***Sociodemographic characteristics***		
Maternal age at birth (years)^b^		
Less than 25	2,274 (14.4)	6,454 (20.4)
25–29	5,457 (34.7)	11,869 (37.5)
30–34	5,320 (33.8)	9,610 (30.3)
35–39	2,296 (14.6)	3,283 (10.4)
40 or more	393 (2.5)	454 (1.4)
Median (IQR) maternal age (years)^b^	30 (26–33)	29 (25–32)
Mother’s country of birth		
UK	14,850 (94.3)	29,870 (94.3)
Non-UK	892 (5.7)	1,802 (5.7)
Marital status/registration type^c^		
Married	13,244 (84.1)	25,792 (81.4)
Joint registration	2,011 (12.8)	4,650 (14.7)
Sole registration	487 (3.1)	1,230 (3.9)
Area deprivation^b^^,^^d^		
1 (Least deprived)	3,016 (19.2)	5,641 (17.9)
2	2,799 (17.8)	5,998 (19.0)
3	2,756 (17.6)	5,871 (18.6)
4	3,285 (20.9)	6,538 (20.7)
5 (Most deprived)	3,830 (24.4)	7,541 (23.9)
Median (IQR) area deprivation^b^^,^^d^	3 (2–4)	3 (2–4)
***Maternal medical and obstetric-related characteristics***		
Previous mode(s) of birth		
Cesarean section(s) only	13,616 (86.5)	19,420 (61.3)
Cesarean section(s) and vaginal birth(s)	2,126 (13.5)	12,252 (38.7)
Parity		
1	8,363 (53.1)	19,260 (60.8)
2 or more	7,379 (46.9)	12,412 (39.2)
Median (IQR) parity	1 (1–2)	1 (1–2)
Interpregnancy interval (months)^b^		
24 or more	6,936 (52)	12,871 (47.9)
12–23	3,965 (29.7)	8,667 (32.2)
Less than 12	2,449 (18.3)	5,340 (19.9)
Median (IQR) interpregnancy interval (months)^b^	25 (15–40)	23 (14–37)
Preexisting or gestational diabetes	388 (2.5)	192 (0.6)
Birth weight (grams)^b^		
Less than 2,500	523 (3.3)	892 (2.8)
2,500–3,999	13,374 (85.4)	26,770 (84.7)
4,000 or more	1,765 (11.3)	3,938 (12.5)
Median (IQR) birth weight (grams)^b^	3,370 (3,060–3,700)	3,430 (3,100–3,758)

^a^ Percentage of those with complete data.

^b^ Missing data: maternal age 4 (0.01%); area deprivation 139 (0.29%); interpregnancy interval 7,186 (15.16%); birth weight 152 (0.32%). Overall, 15.6% of the study population has missing data for 1 or more of these variables.

^c^ Joint registration—parents unmarried but both parents are registered on the birth certificate; sole registration—only the mother is registered on the birth certificate.

^d^ Postcode of residence recorded at the time of birth in question was registered was used to derive Carstairs scores, a measure of area deprivation derived from Census data.

ERCS, elective repeat cesarean section; IQR, interquartile range; VBAC, vaginal birth after previous cesarean.

The median follow-up time was 22.1 years (IQR 4.9 to 27.4 years) and the median maternal age at the end of follow-up was 51 years (IQR 34 to 57 years). During a total of 860,004 person-years of follow-up, 1,159 (2.44%) of the study population had some type of pelvic floor surgery (crude incidence rate per 1,000 person-years 1.35, 95% CI 1.27 to 1.43). A total of 613 (1.29%) of the study population had surgical treatment for pelvic organ prolapse, 531 (1.12%) had surgical treatment for urinary incontinence (472 for stress urinary incontinence alone, 43 for urge urinary incontinence alone, and 16 for mixed urinary incontinence), and 128 (0.27%) had surgical treatment for rectal prolapse or fecal incontinence. The median time between giving birth and undergoing any pelvic floor surgery was 15.4 years (IQR 8.9 to 21.0 years) and the median maternal age at the time of having the surgery was 46 years (IQR 39 to 51).

### Planned VBAC compared to ERCS

**Fig 2:**
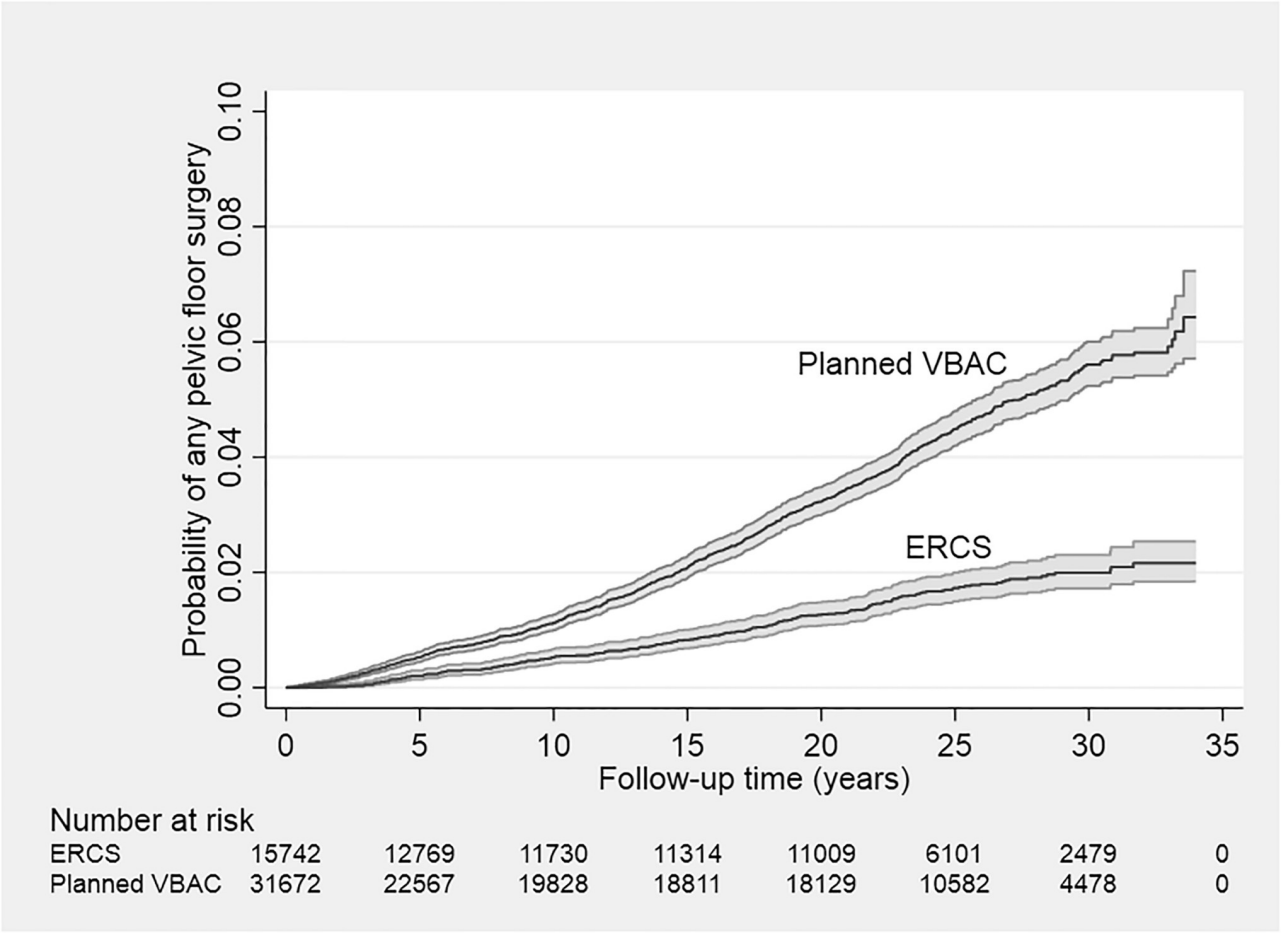
Kaplan–Meier failure curve showing the cumulative probability of any pelvic floor surgery according to planned mode of birth after previous cesarean section. Shaded areas denote 95% CIs. CI, confidence interval; ERCS, elective repeat cesarean section; VBAC, vaginal birth after previous cesarean.

**Fig 3:**
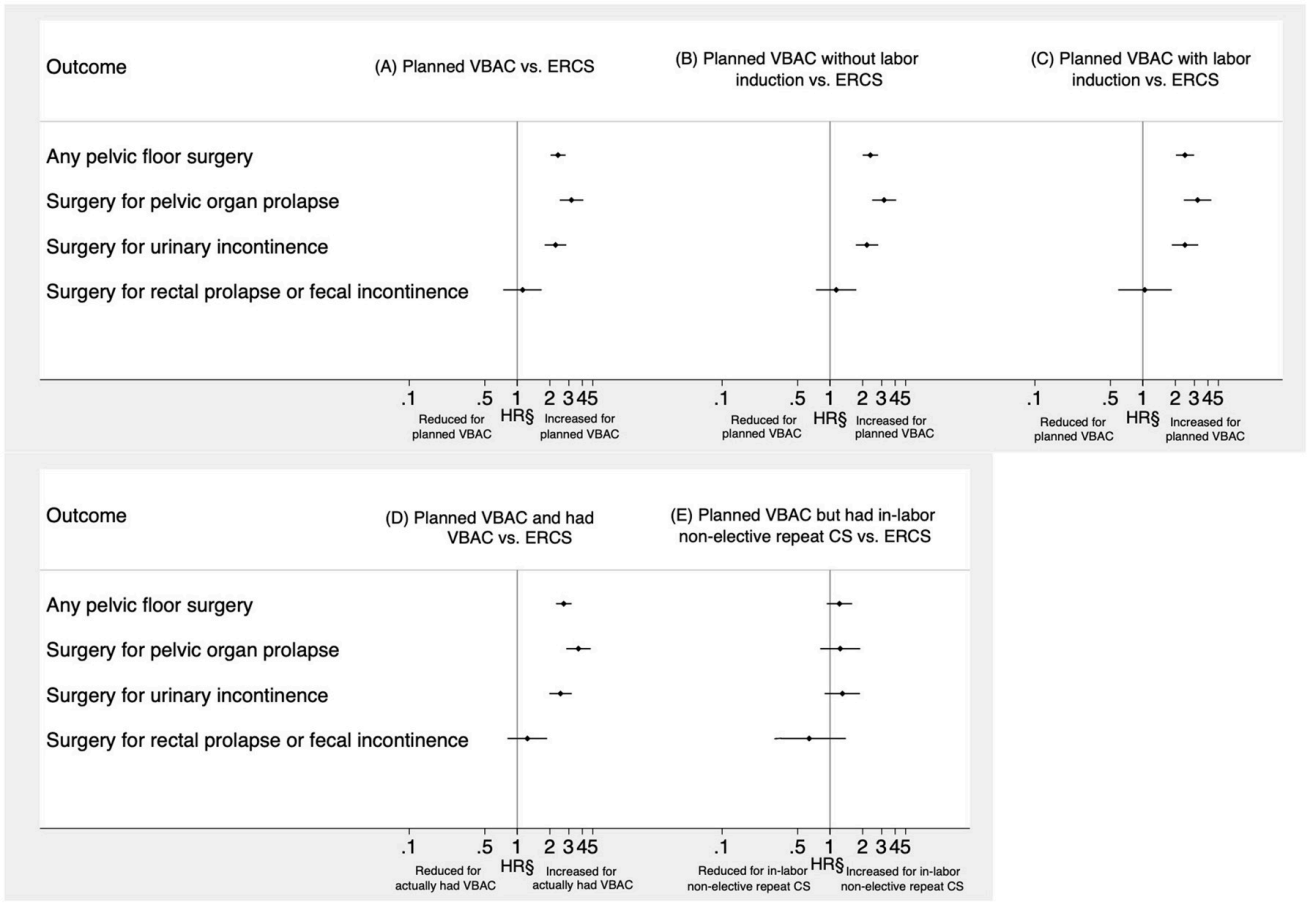
Outcomes following (A) planned VBAC compared to ERCS (B) planned VBAC without labor induction compared to ERCS (C) planned VBAC with labor inuction compared to ERCS (D) planned and actually had VBAC compared to ERCS, (E) planned VBAC but had in-labor non-elective repeat cesarean section compared to ERCS. ^§^Adjusted for year of birth and sociodemographic factors (maternal age, mother’s country of birth, marital status, and area deprivation) and maternal medical and obstetric-related factors (previous mode(s) of birth—categorized as cesarean section only/cesarean section and vaginal birth(s), parity, interpregnancy interval, diabetes, birth weight of child in pregnancy/birth in question treated as a continuous variable). CS, cesarean section; ERCS, elective repeat cesarean section; HR, hazard ratio; VBAC, vaginal birth after previous cesarean.

The crude incidence rate of any type of pelvic floor surgery was 1.75 (95% CI 1.64 to 1.86) per 1,000 person-years in the planned VBAC group and 0.66 (95% CI 0.57 to 0.75) per 1,000 person-years in the ERCS group. During the follow-up period, the probability of having any pelvic floor surgery was consistently higher in the planned VBAC compared to the ERCS group (Fig 2). Having only adjusted for year of birth, women who planned a VBAC had a significantly higher risk than those giving birth by ERCS of undergoing any type of pelvic floor surgery (HR 2.70, 95% CI 2.32 to 3.14) and specific types of pelvic floor (surgery for pelvic organ prolapse: HR 3.80, 95% CI 3.00 to 4.80; surgery for urinary incontinence: HR 2.51, 95% CI 2.02 to 3.12) except surgery for rectal prolapse or fecal incontinence (Table 2). Adjustment for sociodemographic factors had little material impact on the effect estimates, and while further adjustment for maternal medical and obstetric-related factors slightly attenuated all effect estimates, the risk of undergoing some type of pelvic floor surgery (aHR 2.38, 95% CI 2.03 to 2.80), surgery for pelvic organ prolapse (aHR 3.17, 95% CI 2.47 to 4.09), or urinary incontinence (aHR 2.26, 95% CI 1.79 to 2.84) remained significantly higher in women who planned a VBAC compared to those who had an ERCS (Fig 3A). The PAFs associated with planned VBAC were 47.7% (95% CI 39.4% to 55.1%) for any pelvic floor surgery, 59.4% (95% CI 48.3% to 68.5%) for pelvic organ prolapse surgery, and 45.3% (95% CI 32.6% to 56.0%) for urinary incontinence surgery. In the fully adjusted model, there was evidence (*p* = 0.002) that the effect of planned VBAC compared to ERCS on the women’s risk of undergoing surgery for pelvic organ prolapse was greater in women without any prior vaginal births (aHR 4.20, 95% CI 3.05 to 5.78) than in women with 1 or more prior vaginal births (aHR 1.96, 95% CI 1.37 to 2.81). There was also some evidence (*p* = 0.035) that the independent effect of planned VBAC compared to ERCS on the women’s risk of undergoing surgery for pelvic organ prolapse was greater in women with a parity of 1 (aHR 3.80, 95% CI 2.58 to 5.58) than in those with a parity of 2 or more (aHR 2.19, 95% CI 1.55 to 3.08) in the fully adjusted model. Overall, however, the crude incidence rate of pelvic organ prolapse surgery in the study population was higher in women with any prior vaginal births than in those without any prior vaginal births (1.30, 95% CI 1.17 to 1.45 versus 0.45, 95% CI 0.39 to 0.50 per 1,000 person-years) and was higher in women with a parity of 2 or more than in those with a parity of 1 (0.95, 95% CI 0.85 to 1.05 versus 0.52, 95% CI 0.46 to 0.59 per 1,000 person-years). No other significant interactions were found between planned mode of birth and parity or whether the woman had any prior vaginal births.

**Table 2:** Outcomes following planned VBAC compared to ERCS.

Outcomes	ERCS number of events/person-years (rate per 100 person-years)	Planned VBAC number of events/person-years (rate per 100 person-years)	Unadjusted model HR (95% CI)	Base model^1^ HR (95% CI)	Model A^2^ HR (95% CI)	Model B^3^ HR (95% CI)
Any pelvic floor surgery	206/313,960 (0.66)	953/546,044 (1.75)	**2.70 (2.32–3.14)**	**2.70 (2.32–3.14)**	**2.78 (2.39–3.23)**	**2.38 (2.03–2.80)**
			***P* < 0.001**	***P* < 0.001**	***P* < 0.001**	***P* < 0.001**
Surgery for pelvic organ prolapse	81/315,369 (0.26)	532/551,210 (0.97)	**3.80 (3.01–4.81)**	**3.80 (3.00–4.80)**	**3.95 (3.12–5.00)**	**3.17 (2.47–4.09)**
			***P* < 0.001**	***P* < 0.001**	***P* < 0.001**	***P* < 0.001**
Surgery for urinary incontinence	100/315,399 (0.32)	431/552,036 (0.78)	**2.51 (2.02–3.12)**	**2.51 (2.02–3.12)**	**2.58 (2.07–3.21)**	**2.26 (1.79–2.84)**
			***P* < 0.001**	***P* < 0.001**	***P* < 0.001**	***P* < 0.001**
Surgery for rectal prolapse or fecal incontinence	42/316,011 (0.13)	86/556,166 (0.15)	1.18 (0.82–1.70)	1.18 (0.82–1.71)	1.18 (0.82–1.72)	1.12 (0.74–1.68)
			*P* = 0.382	*P* = 0.369	*P* = 0.377	*P* = 0.597

^1^ Base model adjusted for year of birth only.

^2^ Model A adjusted for year of birth and sociodemographic factors (maternal age, mother’s country of birth, marital status, and area deprivation).

^3^ Model B adjusted for variables in Model A and additionally adjusted for maternal medical and obstetric-related factors (previous mode(s) of birth—categorized as cesarean section only/cesarean section and vaginal birth(s), parity, interpregnancy interval, diabetes, birth weight of child in pregnancy/birth in question treated as a continuous variable).

Bold text indicates statistically significant findings at the 5% level.

CI, confidence interval; ERCS, elective repeat cesarean section; HR, hazard ratio; VBAC, vaginal birth after previous cesarean.

### Planned VBAC with and without labor induction compared to ERCS

Of the women who planned a VBAC, 15.7% (7,421/47,410) had their labor induced (16.1% using artificial rupture of membranes alone, 28.1% using oxytocics and/or prostaglandins alone and 55.7% using a combination of these methods). Compared to the women who had an ERCS, both the women who planned a VBAC with and those who planned a VBAC without labor induction had a significantly increased risk of undergoing any pelvic floor surgery, surgery for pelvic organ prolapse, or urinary incontinence (Table 3 and Fig 3B and 3C), mirroring the findings reported for planned VBAC compared to ERCS.

**Table 3:** Outcomes following planned VBAC with and without labor induction compared to ERCS.

Outcomes	ERCS	Planned VBAC without labor induction					Planned VBAC with labor induction				
	Number of events/person-years (rate per 100 person-years)	Number of events/person-years (rate per 100 person-years)	Unadjusted model HR (95% CI)	Base model^1^ HR (95% CI)	Model A^2^ HR (95% CI)	Model B^3^ HR (95% CI)	Number of events/person-years (rate per 100 person-years)	Unadjusted model HR (95% CI)	Base model^1^ HR (95% CI)	Model A^2^ HR (95% CI)	Model B^3^ HR (95% CI)
Any pelvic floor surgery	206/313,960	716/417,741	**2.65**	**2.65**	**2.73**	**2.36**	237/128,292	**2.86**	**2.85**	**2.92**	**2.45**
	(0.66)	(1.71)	**(2.27–3.10)**	**(2.27–3.10)**	**(2.34–3.19)**	**(2.00–2.78)**	(1.85)	**(2.37–3.44)**	**(2.37–3.44)**	**(2.42–3.52)**	**(2.02–2.98)**
			***P* < 0.001**	***P* < 0.001**	***P* < 0.001**	***P* < 0.001**		***P* < 0.001**	***P* < 0.001**	***P* < 0.001**	***P* < 0.001**
Surgery for pelvic organ prolapse	81/315,369	400/421,579	**3.74**	**3.74**	**3.90**	**3.16**	132/129,621	**4.00**	**3.99**	**4.13**	**3.21**
	(0.26)	(0.95)	**(2.95–4.75)**	**(2.94–4.75)**	**(3.06–4.95)**	**(2.45–4.09)**	(1.02)	**(3.04–5.28)**	**(3.02–5.26)**	**(3.13–4.45)**	**(2.39–4.30)**
			***P* < 0.001**	***P* < 0.001**	***P* < 0.001**	***P* < 0.001**		***P* < 0.001**	***P* < 0.001**	***P* < 0.001**	***P* < 0.001**
Surgery for urinary incontinence	100/315,399	319/422,298	**2.42**	**2.43**	**2.50**	**2.19**	112/129,727	**2.78**	**2.78**	**2.84**	**2.45**
	(0.32)	(0.76)	**(1.94–3.03)**	**(1.94–3.04)**	**(2.00–3.13)**	**(1.73–2.79)**	(0.86)	**(2.12–3.64)**	**(2.12–3.64)**	**(2.17–3.71)**	**(1.85–3.25)**
			***P* < 0.001**	***P* < 0.001**	***P* < 0.001**	***P* < 0.001**		***P* < 0.001**	***P* < 0.001**	***P* < 0.001**	***P* < 0.001**
Surgery for rectal prolapse or fecal incontinence	42/316,011	67/425,218	1.20	1.21	1.20	1.14	19/130,938	1.10	1.11	1.11	1.04
	(0.13)	(0.16)	(0.82–1.77)	(0.82–1.77)	(0.82–1.78)	(0.74–1.75)	(0.15)	(0.64–1.90)	(0.65–1.91)	(0.65–1.91)	(0.59–1.85)
			*P* = 0.350	*P* = 0.340	*P* = 0.350	*P* = 0.547		*P* = 0.721	*P* = 0.700	*P* = 0.702	*P* = 0.885

^1^ Base model adjusted for year of birth only.

^2^ Model A adjusted for year of birth and sociodemographic factors (maternal age, mother’s country of birth, marital status, and area deprivation).

^3^ Model B adjusted for variables in Model A and additionally adjusted for maternal medical and obstetric-related factors (previous mode(s) of birth—categorized as cesarean section only/ cesarean section and vaginal birth(s), parity, interpregnancy interval, diabetes, birth weight of child in pregnancy/birth in question treated as a continuous variable).

Bold text indicates statistically significant findings at the 5% level.

CI, confidence interval; ERCS, elective repeat cesarean section; HR, hazard ratio; VBAC, vaginal birth after previous cesarean.

### Actual VBAC and in-labor non-elective repeat cesarean section compared to ERCS

Of the women who planned a VBAC, 84.2% (26,671/31,672) gave birth vaginally as planned (median duration of labor 7 hours, IQR 4 to 10 hours) and 15.8% (5,001/31,672) went on to have an in-labor non-elective repeat cesarean section (median duration of labor 8 hours, IQR 5 to 11 hours). Compared to the women who had an ERCS, only the women who actually had a successful VBAC had an increased risk of undergoing any pelvic floor surgery, surgery for pelvic organ prolapse, or urinary incontinence. The women who went on to have an in-labor non-elective repeat cesarean section had a comparable risk to those who had an ERCS of having pelvic floor surgery (Table 4 and Fig 3D and 3E).

**Table 4:** Outcomes according to actual mode of birth—Planned VBAC and had a VBAC and planned VBAC but had in-labor non-elective repeat cesarean section compared to ERCS.

Outcomes	ERCS	Planned VBAC and had VBAC					Planned VBAC but had in-labor non-elective repeat cesarean section				
	Number of events/person-years (rate per 100 person-years)	Number of events/person-years (rate per 100 person-years)	Unadjusted model HR (95% CI)	Base model^1^ HR (95% CI)	Model A^2^ HR (95% CI)	Model B^3^ HR (95% CI)	Number of events/person-years (rate per 100 person-years)	Unadjusted model HR (95% CI)	Base model^1^ HR (95% CI)	Model A^2^ HR (95% CI)	Model B^3^ HR (95% CI)
Any pelvic floor surgery	206/313,960	880/452,902	**3.01**	**3.01**	**3.10**	**2.69**	73/93,142	1.20	1.20	1.23	1.22
	(0.66)	(1.94)	**(2.59–3.51)**	**(2.59–3.50)**	**(2.66–3.61)**	**(2.28–3.18)**	(0.78)	(0.92–1.57)	(0.92–1.57)	(0.94–1.61)	(0.93–1.60)
			***P* < 0.001**	***P* < 0.001**	***P* < 0.001**	***P* < 0.001**		*P* = 0.176	*P* = 0.180	*P* = 0.130	*P* = 0.150
Surgery for pelvic organ prolapse	81/315,369	503/457,528	**4.34**	**4.33**	**4.52**	**3.68**	29/93,682	1.21	1.21	1.25	1.24
	(0.26)	(1.10)	**(3.43–5.49)**	**(3.42–5.48)**	**(3.57–5.72)**	**(2.84–4.78)**	(0.31)	(0.79–1.85)	(0.79–1.84)	(0.82–1.91)	(0.81–1.90)
			***P* < 0.001**	***P* < 0.001**	***P* < 0.001**	***P* < 0.001**		*P* = 0.381	*P* = 0.390	*P* = 0.308	*P* = 0.328
Surgery for urinary incontinence	100/315,399	393/458,473	**2.76**	**2.76**	**2.84**	**2.51**	38/93,562	1.30	1.30	1.33	1.30
	(0.32)	(0.86)	**(2.21–3.43)**	**(2.21–3.43)**	**(2.28–3.54)**	**(1.98–3.19)**	(0.41)	(0.89–1.88)	(0.89–1.88)	(0.91–1.93)	(0.89–1.89)
			***P* < 0.001**	***P* < 0.001**	***P* < 0.001**	***P* < 0.001**		*P* = 0.172	*P* = 0.172	*P* = 0.139	*P* = 0.173
Surgery for rectal prolapse or fecal incontinence	42/316,011	118/462,261	1.29	1.29	1.29	1.24	11/93,905	0.64	0.65	0.65	0.64
	(0.13)	(0.26)	(0.89–1.87)	(0.89–1.88)	(0.89–1.89)	(0.81–1.89)	(0.12)	(0.30–1.37)	(0.30–1.38)	(0.30–1.38)	(0.30–1.40)
			*P* = 0.185	*P* = 0.177	*P* = 0.182	*P* = 0.317		*P* = 0.253	*P* = 0.259	*P* = 0.258	*P* = 0.267

^1^ Base model adjusted for year of birth only.

^2^ Model A adjusted for year of birth and sociodemographic factors (maternal age, mother’s country of birth, marital status, and area deprivation).

^3^ Model B adjusted for variables in Model A and additionally adjusted for maternal medical and obstetric-related factors (previous mode(s) of birth—categorized as cesarean section only/cesarean section and vaginal birth(s), parity, interpregnancy interval, diabetes, birth weight of child in pregnancy/birth in question treated as a continuous variable).

Bold text indicates statistically significant findings at the 5% level.

CI, confidence interval; ERCS, elective repeat cesarean section; HR, hazard ratio; VBAC, vaginal birth after previous cesarean.

### Sensitivity analyses

The complete case analyses (Tables C–E in S2 File), the analyses conducted in the subgroup of women who had all their previous births captured in the dataset (33,248, Tables I–K in S2 File), and the analyses defining planned VBAC as birth vaginally or by non-elective cesarean section with a duration of labor of >4 hours rather than ≥1 hour (Table M in S2 File) resulted in similar effect estimates. The effect estimates from the analyses conducted in the subgroup of women who gave birth between 1 January 1983 and 31 December 1986 (12,699, median follow-up time 30.3 years, IQR 4.3 to 31.9, median maternal age at end of follow-up 56 years, IQR 32 to 61 years, Table L in S2 File) were smaller although still in the range of those reported for the main analysis with the same significant associations seen. The E-values suggest that strong unmeasured confounding would be needed to explain the observed associations between the exposures and outcomes (Tables F–H in S2 File). For example, to fully explain the observed HR of 2.38 for the association between planned mode of birth after previous cesarean and any type of pelvic floor surgery (Table 2), an unmeasured confounder would need to be associated with at least a 4.19-fold increased risk of both the primary exposure and outcome through pathways independent of the covariates included in the fully adjusted model; to move the lower CI to include the null, an unmeasured confounder would need to be associated with at least a 3.48-fold increased risk of both the primary exposure and outcome, above and beyond the measured covariates.

## Discussion

This population-based cohort study of 47,414 singleton term births to women with 1 or more previous cesarean sections found that planned VBAC compared to ERCS was associated with a greater than 2-fold increased risk of the woman undergoing any type of pelvic floor surgery and a 2- to 3-fold increased risk of the woman having surgery for pelvic organ prolapse or urinary incontinence. Assuming the assumptions underliying the PAF such as causality are met [42], an estimated 48% of pelvic floor surgeries could be avoided if women had an ERCS rather than a planned VBAC. The increased risks seen in the planned VBAC group were only apparent in the women who actually gave birth vaginally as planned, with the women who needed an in-labor non-elective repeat cesarean section having a comparable risk of pelvic floor surgery to those who had an ERCS. However, the absolute risk of undergoing pelvic floor surgery during the study follow-up period was small regardless of the planned or actual mode of birth, noting the relatively young median maternal age at the end of follow-up (51 years in the main analysis). Overall, the incidence rate of any pelvic floor surgery was 1.75 per 1,000 person-years in the planned VBAC group and 0.66 per 1,000 person years in the ERCS group.

To the best of our knowledge, this is the first study to examine the possible effects of planned mode of birth after previous cesarean section on the woman’s long-term risk of experiencing adverse pelvic floor outcomes. Consistent with the findings of our study, a recent review of systematic reviews [27] reported that the available evidence suggests that the risk of urinary incontinence and pelvic organ prolapse is increased with vaginal birth compared with birth by cesarean section, while most of the existing evidence shows no significant association between mode of birth and subsequent risk of fecal incontinence. However, the review highlights several significant limitations with the existing evidence including the possibility of selection bias and the fact that most studies did not account for important potential confounding factors or differentiate between pre-labor and in-labor cesarean section. Another recent review [43], consistent with our findings, reported that the available evidence suggests that among primiparous women, elective cesarean section is protective against urinary incontinence when compared to vaginal birth. However, in contrast to our findings, this review also concluded that among primiparous women, elective cesarean section may be protective against urinary incontinence when compared with in-labor cesarean section and is associated with a decreased risk of fecal or anal incontinence compared with vaginal birth. The review concluded that there was not enough evidence to assess the effect of elective cesarean birth on pelvic organ prolapse. Our study suggests ERCS is protective against urinary incontinence and pelvic organ prolapse when compared with either planned VBAC or actual VBAC, but not when compared with in-labor non-elective repeat cesarean section. These findings highlight the role of the passage of the fetus through the birth canal rather than labor in pelvic floor dysfunction requiring surgery. The overall incidence rate of pelvic organ prolapse surgery in our cohort was higher among women with compared to without any prior vaginal births and in those with a parity of 2 or more compared to 1, supporting previous literature suggesting these are risk factors for pelvic organ prolapse [44]. The effect of planned VBAC compared to ERCS on the women’s risk of undergoing surgery for pelvic organ prolapse, however, was found in our study to be greater among women without than with any prior vaginal births and in those with a parity of 1 compared to 2 or more. This may indicate that it is the first vaginal birth that has the greatest impact on the pelvic floor and the subsequent risk of pelvic floor surgery, acknowleding that we did not have the numbers to stratify the analysis by number of prior vaginal births.

Despite the relatively long duration of follow-up, particularly in comparison to much of the existing literature [27], most women in our study were still relatively young at the end of follow-up (median age 51 years, IQR 34 to 57 years in the main analysis) and the rates of pelvic floor surgery observed were lower than one would expect had the study included older women [21,45]. Data from several high-income settings suggest that while surgery for urinary incontinence tends to be most commonly performed in women in their fifties, pelvic organ prolapse and rectal prolapse surgery is most commonly performed when women are in their sixties or seventies [46–51]. While the observed rates of pelvic floor surgery are comparable to some reported incidence rates in non-elderly (less than 60 or 65 years old) women [49,52,53], other studies [22,54] have observed higher incidence rates in non-elderly women. Differences in the rates and patterns of risk factors between populations, variation in treatment-seeking behavior, what treatment is offered or accepted, and methodological differences such as the use of different data coding schemes may explain these differences.

Strengths of this study include its large population-based design with a relatively long duration of follow-up and the use of prospectively collected routine data that is subject to regular quality checks. Another key strength is the fact that the study was confined to women considered clinically eligible to plan VBAC based on UK guidelines [4,5]. We were also able to examine the influence of many a priori potential confounders, particularly in the sensitivity analysis confined to women who had all their previous births captured in the dataset. Nevertheless, the possibility of residual confounding due to mis-measured or unmeasured confounding factors that we have not been able to consider, such as maternal BMI and smoking status that were not recorded during the study time period, remains. Also, although we excluded births to women with any evidence of pelvic floor surgery before they gave birth, this would not have excluded those with previous/preexisting pelvic floor disorders not managed surgically that could have led to residual confounding if the presence of such pelvic floor disorders before birth influenced women’s planned mode of birth. However, the calculated E-values suggest strong unmeasured confounding would be needed to fully explain the observed associations between the exposures and outcomes. It is also worth pointing out that in the absence of being able to conduct a large randomized controlled trial, which a previous study suggests is unlikely to be feasible [11], large observational population-based studies such as this study offer the best chance of improving the evidence base in this area.

We acknowledge that the criteria used to define planned mode of birth could misclassify women who planned ERCS but went into labor before their scheduled cesarean date, noting that the sensitivity analysis defining planned VBAC using slightly different criteria resulted in similar effect estimates. We also acknowledge the possibility that some misclassification of the other variables of interest may have occurred, with the completeness and accuracy of diagnostic and procedure codes often a particular concern with routine data. Such misclassification may have potentially biased the study findings towards the null or under- or overestimated effect estimates depending upon whether any misclassification was random or systematic in nature, respectively. However, the completeness and quality of most routinely collected Scottish health data is considered to be very high, with some of the data sources containing statutorily collected data and much of the data subject to regular quality checks as summarized in Table A in S2 File. Exclusion of pre-labor or missing labor non-elective cesarean sections could be viewed as another limitation in the sense that women may make a decision about planned VBAC or ERCS many weeks before term and this preference may possibly influence management if a woman attends with a “non-elective” indication for delivery. However, pre-labor non-elective cesarean sections were excluded on the presumption that this situation removes the option of choice about whether to have a planned VBAC or ERCS. We also excluded births by non-elective cesarean section missing information about duration of labor, as we could not tell whether these non-elective cesarean sections were carried out prior to or during labor. In any case, only a relatively small number of women were excluded for these reasons (as shown in Fig 1, of the 61,594 term births to women with ≥1 previous cesarean sections identified, 2,632 were births by pre-labor non-elective cesarean section and 927 were non-elective cesarean sections missing duration of labor). We also recognize that by looking at pelvic floor surgery, we are likely to have only captured more severe cases of pelvic floor disorders and would not have captured cases managed with non-surgical interventions such as pessaries and/or women who were not considered fit for surgery. However, we have no reason to believe that our outcome ascertainment would have been dependent on the exposures of interest to have potentially biased the findings. We were also not able to investigate the risk of pelvic floor surgery according to the stage of labor when a non-elective cesarean section was performed in a VBAC attempt, as this information is not captured in the routinely collected Scottish datasets. We also did not have the power to examine surgery for rectal prolapse and surgery for fecal incontinence as 2 separate outcomes. While missing covariate data is recognized as another limitation, our use of multiple imputation is regarded as a valid approach for dealing with this issue assuming the data are missing at random [55]. Also, although the main analyses were prespecified based on clear hypotheses and biological plausibility, the performance of multiple comparisons would have increased the chance of type 1 errors. However, it is worth noting that using a more stringent *p*-value to allow for multiple testing would not have altered the main findings. Finally, to allow at least 20 years of follow-up our study included births between 1983 and 1996 and we recognize that there have been changes in the characteristics/risk profile of the obstetric population since this time such as increasing maternal age [1,56,57]. However, our analyses were adjusted for many of these factors, so the reported relative effect estimates are likely to be generalizable to more contemporary obstetric populations of women giving birth to a singleton at term after previous cesarean section in high-income settings.

## Conclusions and implications

This study suggests that among women with previous cesarean section giving birth to a singleton at term, planned VBAC compared to ERCS is associated with an increased risk of the woman subsequently undergoing pelvic floor surgery including surgery for pelvic organ prolapse and urinary incontinence. However, these risks appear to be only apparent in women who actually give birth vaginally as planned, highlighting the role of vaginal birth rather than labor in pelvic floor dysfunction requiring surgery. These findings provide useful additional information to counsel the increasing numbers of women with previous cesarean section about the risks and benefits associated with their future birth choices, as recommended by clinical guidelines [4–8].

## Supporting information

S1 File Record checklist. (DOCX)

S2 File Supporting file. Fig A. Schematic to explain time-varying exposure model that propose to use, allowing inclusion of more than 1 birth per woman in the study cohort. Text A. Extract from application to the Public Benefit and Privacy Panel for Health and Social Care Scotland, taken from application submitted in March 2018. Table A. Data sources. Table B. Data sources, codes, and database fields used to identify study population, exposures, outcomes, and covariates. Table C. Complete case analysis of outcomes following planned VBAC compared to ERCS. Table D. Complete case analysis of outcomes following planned VBAC with and without labor induction compared to ERCS. Table E. Complete case analysis of outcomes according to actual mode of birth—planned VBAC and had a VBAC and planned VBAC but had in-labor non-elective repeat cesarean section compared to ERCS. Table F. E-values for the observed associations between planned mode of birth after previous cesarean section (planned VBAC vs. ERCS) and pelvic floor outcomes. Table G. E-values for the observed associations between planned mode of birth after previous cesarean section (planned VBAC with and without labor induction vs. ERCS) and pelvic floor outcomes. Table H. E-values for the observed associations between actual mode of birth after previous cesarean section (planned VBAC and had a VBAC and planned VBAC but had in-labor non-elective repeat cesarean section compared vs. ERCS) and pelvic floor outcomes. Table I. Outcomes following planned VBAC compared to ERCS in women who had all their previous births in the SMR02. Table J. Outcomes following planned VBAC with and without labor induction compared to ERCS in women who had all their previous births in the SMR02. Table K. Outcomes according to actual mode of birth—planned VBAC and had a VBAC and planned VBAC but had in-labor non-elective repeat cesarean section compared to ERCS in women who had all their previous births in the SMR02. Table L. Rate and hazard ratio of any pelvic floor surgery by the exposures of interest in the subgroup of women who gave birth between 1 January 1983 and 31 December 1986. Table M. Outcomes following planned VBAC compared to ERCS, defining planned VBAC as birth vaginally or by non-electve cesarean section with a duration of labor of >4 hours (rather than ≥1 hour).
(DOCX)

## Abbreviations

aHR: adjusted hazard ratio
CI: confidence interval
ERCS: elective repeat cesarean section
HR: hazard ratio
IQR: interquartile range
NIHR: National Institute for Health and Care Research
PAF: population attributable fraction
VBAC: vaginal birth after previous cesarean

## References

[1] Public Health Scotland. Births in Scottish Hospitals Year ending 31 March 2020. Edinburgh: 2020.

[2] NHS Digital. NHS Maternity Statistics, England 2019–20. 2020.

[3] Welsh Government. Maternity and birth statistics, Wales 2020. 2020.

[4] Royal College of Obstetricians and Gynaecologists. Birth After Previous Caesarean Birth, Green-top Guideline No. 45. London, 2015.

[5] National Institute for Health and Clinical Excellence. Caesarean section NICE clinical guideline 132. 2011. 31886991

[6] American College of Obstetricians and Gynecologists. ACOG Practice Bulletin No. 205: Vaginal Birth After Cesarean Delivery. Obstet Gynecol. 2019;133(2):e110–e127. doi: 10.1097/AOG.0000000000003078 30681543

[7] The Royal Australian and New Zealand College of Obstetricians and Gynaecologists. Birth after previous caesarean section. 2015.

[8] Martel MJ, MacKinnon CJ, Clinical Practice Obstetrics Committee SoO, Gynaecologists of C. Guidelines for vaginal birth after previous Caesarean birth. J Obstet Gynaecol Can. 2005;27(2):164–88. doi: 10.1016/s1701-2163(16)30188-8 . 15943001

[9] Fitzpatrick KE, Kurinczuk JJ, Bhattacharya S, Quigley MA. Planned mode of delivery after previous cesarean section and short-term maternal and perinatal outcomes: A population-based record linkage cohort study in Scotland. PLoS Med. 2019;16(9):e1002913. Epub 2019/09/25. doi: 10.1371/journal.pmed.1002913 . 31550245 PMC6759152

[10] Guise J-M, Eden K, Emeis C, Denman MA, Marshall N, Fu RR, et al. Vaginal birth after cesarean: new insights. Evid Rep Technol Assess (Summ). 2010;191:1–397. PMC4781304 20629481

[11] Crowther CA, Dodd JM, Hiller JE, Haslam RR, Robinson JS. Planned vaginal birth or elective repeat caesarean: Patient preference restricted cohort with nested randomised trial. PLoS Med. 2012;9(3):e1001192. doi: 10.1371/journal.pmed.1001192 22427749 PMC3302845

[12] Kok N, Ruiter L, Lindeboom R, de Groot C, Pajkrt E, Mol BW, et al. Elective repeat cesarean delivery compared with trial of labor after a prior cesarean delivery: a propensity score analysis. Eur J Obstet Gynecol Reprod Biol. 2015;195:214–218. doi: 10.1016/j.ejogrb.2015.09.011 26599733

[13] Fitzpatrick KE, Kurinczuk JJ, Quigley MA. Planned mode of birth after previous caesarean section and special educational needs in childhood: a population-based record linkage cohort study. BJOG. 2021;128(13):2158–68. Epub 2021/07/04. doi: 10.1111/1471-0528.16828 . 34216080 PMC9291107

[14] Fitzpatrick KE, Quigley MA, Kurinczuk JJ. Planned mode of birth after previous cesarean section: A structured review of the evidence on the associated outcomes for women and their children in high-income setting. Front Med (Lausanne). 2022;9:920647. Epub 2022/09/24. doi: 10.3389/fmed.2022.920647 . 36148449 PMC9486480

[15] Nygaard I, Barber MD, Burgio KL, Kenton K, Meikle S, Schaffer J, et al. Prevalence of symptomatic pelvic floor disorders in US women. JAMA. 2008;300(11):1311–6. Epub 2008/09/19. doi: 10.1001/jama.300.11.1311 . 18799443 PMC2918416

[16] Zeleke BM, Bell RJ, Billah B, Davis SR. Symptomatic pelvic floor disorders in community-dwelling older Australian women. Maturitas. 2016;85:34–41. Epub 2016/02/10. doi: 10.1016/j.maturitas.2015.12.012 . 26857877

[17] Hannestad YS, Rortveit G, Sandvik H, Hunskaar S, Norwegian EsEoIitCoN-T. A community-based epidemiological survey of female urinary incontinence: the Norwegian EPINCONT study. Epidemiology of Incontinence in the County of Nord-Trondelag. J Clin Epidemiol. 2000;53(11):1150–7. Epub 2000/12/07. doi: 10.1016/s0895-4356(00)00232-8 . 11106889

[18] Walker GJ, Gunasekera P. Pelvic organ prolapse and incontinence in developing countries: review of prevalence and risk factors. Int Urogynecol J. 2011;22(2):127–35. Epub 2010/07/10. doi: 10.1007/s00192-010-1215-0 . 20617303

[19] Meyer I, Richter HE. Impact of fecal incontinence and its treatment on quality of life in women. Womens Health (Lond). 2015;11(2):225–38. Epub 2015/03/18. doi: 10.2217/whe.14.66 . 25776296 PMC4394646

[20] Pizzol D, Demurtas J, Celotto S, Maggi S, Smith L, Angiolelli G, et al. Urinary incontinence and quality of life: a systematic review and meta-analysis. Aging Clin Exp Res. 2021;33(1):25–35. Epub 2020/09/24. doi: 10.1007/s40520-020-01712-y . 32964401 PMC7897623

[21] Abdel-Fattah M, Familusi A, Fielding S, Ford J, Bhattacharya S. Primary and repeat surgical treatment for female pelvic organ prolapse and incontinence in parous women in the UK: a register linkage study. BMJ Open. 2011;1(2):e000206. Epub 2011/11/22. doi: 10.1136/bmjopen-2011-000206 . 22102637 PMC3221293

[22] Wu JM, Matthews CA, Conover MM, Pate V, Jonsson Funk M. Lifetime risk of stress urinary incontinence or pelvic organ prolapse surgery. Obstet Gynecol. 2014;123(6):1201–6. Epub 2014/05/09. doi: 10.1097/AOG.0000000000000286 . 24807341 PMC4174312

[23] Weber AM. Elective cesarean delivery: the pelvic perspective. Clin Obstet Gynecol. 2007;50(2):510–7. Epub 2007/05/22. doi: 10.1097/GRF.0b013e31804c9cae . 17513936

[24] Turner CE, Young JM, Solomon MJ, Ludlow J, Benness C. Incidence and etiology of pelvic floor dysfunction and mode of delivery: an overview. Dis Colon Rectum. 2009;52(6):1186–95. Epub 2009/07/08. doi: 10.1007/DCR.0b013e31819f283f . 19581867

[25] Koc O, Duran B. Role of elective cesarean section in prevention of pelvic floor disorders. Curr Opin Obstet Gynecol. 2012;24(5):318–23. Epub 2012/07/21. doi: 10.1097/GCO.0b013e3283573fcb . 22814811

[26] Gachon B, De Tayrac R, Schmitz T, Mahmood T, Nizard J, Fritel X. Should we advise women that pre-labor caesarean section prevents pelvic floor dysfunction? Eur J Obstet Gynecol Reprod Biol. 2020;244:31–4. Epub 2019/11/16. doi: 10.1016/j.ejogrb.2019.10.037 . 31731021

[27] Lopez-Lopez AI, Sanz-Valero J, Gomez-Perez L, Pastor-Valero M. Pelvic floor: vaginal or caesarean delivery? A review of systematic reviews. Int Urogynecol J. 2021;32(7):1663–73. Epub 2020/10/18. doi: 10.1007/s00192-020-04550-8 . 33068134

[28] Hehir MP, Fitzpatrick M, Cassidy M, Murphy M, O’Herlihy C. Are women having a vaginal birth after a previous caesarean delivery at increased risk of anal sphincter injury? BJOG. 2014;121(12):1515–20. Epub 2014/03/14. doi: 10.1111/1471-0528.12706 . 24621202

[29] Jardine JE, Knight HE, Carroll FE, Gurol-Urganci I. Risk of obstetric anal sphincter injury in women having a vaginal birth after a previous caesarean section: A population-based cohort study. Eur J Obstet Gynecol Reprod Biol. 2019;236:7–13. Epub 2019/03/15. doi: 10.1016/j.ejogrb.2019.02.004 . 30870742

[30] D’Souza JC, Monga A, Tincello DG. Risk factors for obstetric anal sphincter injuries at vaginal birth after caesarean: a retrospective cohort study. Int Urogynecol J. 2019;30(10):1747–53. Epub 2019/07/04. doi: 10.1007/s00192-019-03978-x . 31267138 PMC6795633

[31] Huber M, Malers E, Tunon K. Pelvic floor dysfunction one year after first childbirth in relation to perineal tear severity. Sci Rep. 2021;11(1):12560. Epub 2021/06/17. doi: 10.1038/s41598-021-91799-8 . 34131194 PMC8206367

[32] Borello-France D, Burgio KL, Richter HE, Zyczynski H, Fitzgerald MP, Whitehead W, et al. Fecal and urinary incontinence in primiparous women. Obstet Gynecol. 2006;108(4):863–72. Epub 2006/10/03. doi: 10.1097/01.AOG.0000232504.32589.3b . 17012447

[33] LaCross A, Groff M, Smaldone A. Obstetric anal sphincter injury and anal incontinence following vaginal birth: a systematic review and meta-analysis. J Midwifery Womens Health. 2015;60(1):37–47. Epub 2015/02/26. doi: 10.1111/jmwh.12283 . 25712278

[34] Royal College of Obstetricians and Gynaecologists. Late Intrauterine Fetal Death and Stillbirth Green-top Guidelines No.55. London, 2010. 10.1111/1471-0528.17844 39467688

[35] Thom DH, Brown JS, Schembri M, Ragins AI, Creasman JM, Van Den Eeden SK. Parturition events and risk of urinary incontinence in later life. Neurourol Urodyn. 2011;30(8):1456–61. Epub 2011/07/23. doi: 10.1002/nau.21166 . 21780171 PMC3197896

[36] Durnea CM, Khashan AS, Kenny LC, Durnea UA, Dornan JC, O’Sullivan SM, et al. What is to blame for postnatal pelvic floor dysfunction in primiparous women-Pre-pregnancy or intrapartum risk factors? Eur J Obstet Gynecol Reprod Biol. 2017;214:36–43. Epub 2017/05/20. doi: 10.1016/j.ejogrb.2017.04.036 . 28525825

[37] Okeahialam NA, Dworzynski K, Jacklin P, McClurg D, Guideline C. Prevention and non-surgical management of pelvic floor dysfunction: summary of NICE guidance. BMJ. 2022;376:n3049. Epub 2022/01/08. doi: 10.1136/bmj.n3049 . 34992080

[38] Bartlett JW, Seaman SR, White IR, Carpenter JR. Multiple imputation of covariates by fully conditional specification: Accommodating the substantive model. Stat Methods Med Res. 2014;24:462–487. doi: 10.1177/0962280214521348 24525487 PMC4513015

[39] White IR, Royston P. Imputing missing covariate values for the Cox model. Stat Med. 2009;28(15):1982–98. Epub 2009/05/20. doi: 10.1002/sim.3618 . 19452569 PMC2998703

[40] Natarajan S, Lipsitz SR, Rimm E. A simple method of determining confidence intervals for population attributable risk from complex surveys. Stat Med. 2007;26(17):3229–39. Epub 2007/02/20. doi: 10.1002/sim.2779 . 17309113

[41] VanderWeele TJ, Ding P. Sensitivity Analysis in Observational Research: Introducing the E-Value. Ann Intern Med. 2017;167(4):268–74. Epub 2017/07/12. doi: 10.7326/M16-2607 . 28693043

[42] Mansournia MA, Altman DG. Population attributable fraction. BMJ. 2018;360:k757. Epub 2018/02/24. doi: 10.1136/bmj.k757 . 29472187

[43] Tholemeier L, Souders CP, Bresee C, Nik-Ahd F, Caron A, Eilber KS, et al. Seeking the Truth About Primary Elective Cesarean Delivery and Pelvic Floor Disorders: A Systematic Review and Meta-Analysis. Female Pelvic Med Reconstr Surg. 2022;28(3):e108–e14. Epub 2022/03/11. doi: 10.1097/SPV.0000000000001164 . 35272343

[44] Vergeldt TF, Weemhoff M, IntHout J, Kluivers KB. Risk factors for pelvic organ prolapse and its recurrence: a systematic review. Int Urogynecol J. 2015;26(11):1559–73. Epub 2015/05/15. doi: 10.1007/s00192-015-2695-8 . 25966804 PMC4611001

[45] Olsen AL, Smith VJ, Bergstrom JO, Colling JC, Clark AL. Epidemiology of surgically managed pelvic organ prolapse and urinary incontinence. Obstet Gynecol. 1997;89(4):501–6. Epub 1997/04/01. doi: 10.1016/S0029-7844(97)00058-6 . 9083302

[46] Royal College of Obstetricians and Gynaecologists. Hospital Episode Statistics as a source of information on safety and quality in gynaecology to support revalidation. 2012.

[47] Fialkow MF, Newton KM, Lentz GM, Weiss NS. Lifetime risk of surgical management for pelvic organ prolapse or urinary incontinence. Int Urogynecol J Pelvic Floor Dysfunct. 2008;19(3):437–40. Epub 2007/09/27. doi: 10.1007/s00192-007-0459-9 . 17896064

[48] Lowenstein E, Ottesen B, Gimbel H. Incidence and lifetime risk of pelvic organ prolapse surgery in Denmark from 1977 to 2009. Int Urogynecol J. 2015;26(1):49–55. Epub 2014/05/21. doi: 10.1007/s00192-014-2413-y . 24842118

[49] Kurkijarvi K, Aaltonen R, Gissler M, Makinen J. Pelvic organ prolapse surgery in Finland from 1987 to 2009: A national register based study. Eur J Obstet Gynecol Reprod Biol. 2017;214:71–7. Epub 2017/05/12. doi: 10.1016/j.ejogrb.2017.04.004 . 28494266

[50] El-Dhuwaib Y, Pandyan A, Knowles CH. Epidemiological trends in surgery for rectal prolapse in England 2001–2012: an adult hospital population-based study. Colorectal Dis. 2020;22(10):1359–66. Epub 2020/04/30. doi: 10.1111/codi.15094 . 32346972

[51] Haya N, Baessler K, Christmann-Schmid C, de Tayrac R, Dietz V, Guldberg R, et al. Prolapse and continence surgery in countries of the Organization for Economic Cooperation and Development in 2012. Am J Obstet Gynecol. 2015;212(6):755 e1–e27. Epub 2015/03/01. doi: 10.1016/j.ajog.2015.02.017 . 25724403

[52] Wu JM, Gandhi MP, Shah AD, Shah JY, Fulton RG, Weidner AC. Trends in inpatient urinary incontinence surgery in the USA, 1998–2007. Int Urogynecol J. 2011;22(11):1437–43. Epub 2011/10/07. doi: 10.1007/s00192-011-1509-x . 21975533

[53] Kurkijarvi K, Aaltonen R, Gissler M, Makinen J. Surgery for stress urinary incontinence in Finland 1987–2009. Int Urogynecol J. 2016;27(7):1021–7. Epub 2015/12/30. doi: 10.1007/s00192-015-2926-z . 26713330

[54] Smith FJ, Holman CD, Moorin RE, Tsokos N. Lifetime risk of undergoing surgery for pelvic organ prolapse. Obstet Gynecol. 2010;116(5):1096–100. Epub 2010/10/23. doi: 10.1097/AOG.0b013e3181f73729 . 20966694

[55] Nguyen CD, Carlin JB, Lee KJ. Model checking in multiple imputation: an overview and case study. Emerg Themes Epidemiol. 2017;14:8. Epub 2017/08/31. doi: 10.1186/s12982-017-0062-6 . 28852415 PMC5569512

[56] Mathews TJ, Hamilton BE. Mean age of mother, 1970–2000. Natl Vital Stat Rep. 2002;51(1):1–13. Epub 2003/02/05. . 12564162

[57] Breart G. Delayed childbearing. Eur J Obstet Gynecol Reprod Biol. 1997;75(1):71–3. Epub 1998/02/03. doi: 10.1016/s0301-2115(97)00190-5 . 9447350

