# Supplementary material for: Planned mode of birth after previous cesarean section and risk of undergoing pelvic floor surgery: A Scottish population-based record linkage cohort study
Source: PLoS Med. 2022 Nov 22;19(11):e1004119. doi: 10.1371/journal.pmed.1004119 (PMC9681109; doi:10.1371/journal.pmed.1004119)
Supplement: S2 File — Fig A. Schematic to explain time-varying exposure model that propose to use, allowing inclusion of more than 1 birth per woman in the study cohort. Text A. Extract from application to the Public Benefit and Privacy Panel for Health and Social Care Scotland, taken from application submitted in March 2018. Table A. Data sources. Table B. Data sources, codes, and database fields used to identify study population, exposures, outcomes, and covariates. Table C. Complete case analysis of outcomes following planned VBAC compared to ERCS. Table D. Complete case analysis of outcomes following planned VBAC with and without labor induction compared to ERCS. Table E. Complete case analysis of outcomes according to actual mode of birth—planned VBAC and had a VBAC and planned VBAC but had in-labor non-elective repeat cesarean section compared to ERCS. Table F. E-values for the observed associations between planned mode of birth after previous cesarean section (planned VBAC vs. ERCS) and pelvic floor outcomes. Table G. E-values for the observed associations between planned mode of birth after previous cesarean section (planned VBAC with and without labor induction vs. ERCS) and pelvic floor outcomes. Table H. E-values for the observed associations between actual mode of birth after previous cesarean section (planned VBAC and had a VBAC and planned VBAC but had in-labor non-elective repeat cesarean section compared vs. ERCS) and pelvic floor outcomes. Table I. Outcomes following planned VBAC compared to ERCS in women who had all their previous births in the SMR02. Table J. Outcomes following planned VBAC with and without labor induction compared to ERCS in women who had all their previous births in the SMR02. Table K. Outcomes according to actual mode of birth—planned VBAC and had a VBAC and planned VBAC but had in-labor non-elective repeat cesarean section compared to ERCS in women who had all their previous births in the SMR02. Table L. Rate and hazard ratio of any pelvic floor su [file pmed.1004119.s002.docx]

**S2 File**

**Table A. Data sources***

| **Data source** | **Description** |
| --- | --- |
| National Records of  Scotland (NRS) live births and stillbirths | Statutory data on all live births and stillbirths occurring in Scotland, subject to various quality checks [1]. |
| The Scottish Morbidity Record Maternity Inpatient and Day Case dataset (SMR02) | Data on all inpatient & day case discharges from obstetric specialities in National Health Service (NHS) Scotland. SMR02 has had a national coverage of ~98% of all births in NRS since the late 1970s [2], and is subject to regular quality checks [3, 4]. The quality assurance exercises published in 2010 and 2019 found that the key fields used in this study, including mode of birth, matched the information found in the medical records in ~90% or more of the records sampled. |
| The Scottish Morbidity Record General/Acute Inpatient and Day case dataset (SMR01) | Demographic & clinical data on all hospital inpatient & day case discharges from acute specialties in NHS Scotland. Data subject to regular quality assurance checks. Latest quality assurance assessment found that the main condition & main operation/procedure were 89% & 94% accurate respectively [5] |
| NRS deaths | Statutory data about all deaths occurring in Scotland, subject to various quality checks [1]. |
| The Community Health Index (CHI) database | Register of all individuals in NHS Scotland. This will be used to determine dates emigration from Scotland. |

*Linkable data from each of the data sources was available from 1981-2016 at the time it was requested.

**Table B.** **Data sources, codes and database fields used to identify study population, exposures, outcomes, and covariates**

|  | **Data source** | **Database fields/codes** |
| --- | --- | --- |
| **Inclusion criteria** |  |  |
| ≥1 previous cesarean sections | SMR02 | Previous caesarean sections field of pregnancy/birth in question ≥1 OR at least one previous delivery with a code for caesarean section in mode of delivery field (7 or 8) and/or an OPCS-4 or OPCS-3 code for caesarean section (R17-R18, R251, 764-766, 769), using SMR02 records going back as far as 1981 |
| Singleton birth | NRS live births and stillbirths | Pregnancy/birth in question has numbirths field=1 |
| Term birth | SMR02 | Gestation at birth in question 37-41 completed weeks according to estimated gestation field (containing number of completed weeks of gestation as judged by the clinician, usually on the basis of ultrasound) OR according to gestation derived from date of delivery and date of last menstrual period fields if estimated gestation missing (0.02% of eligible births) |
| **Exclusion criteria** |  |  |
| Non-cephalic presentation | SMR02 | Delivery episode of pregnancy/birth in question has a code for breech or shoulder in presentation at delivery field (4 or 6) OR a code for breech delivery or breech extraction in mode of delivery field (5 or 6) OR an ICD-10 or ICD-9 code for breech delivery or breech extraction (O801, O830-O831, 7630) OR an OPCS-4 or OPCS-3 code for breech delivery or breech extraction (R19-R20, 757, 762) OR an ICD-10 or ICD-9 code for maternal care for malpresentation of fetus (0320-0322, 0326-0329, 6520, 6522, 6523, 6527-6529) |
| Placenta praevia | SMR02 | Delivery episode of pregnancy/birth in question has an ICD-10 or ICD-9 code for placenta praevia (O440-O441, 6410-6411) |
| Abdominal pregnancy | SMR02 | Delivery episode of pregnancy/birth in question has an ICD-10 code for delivery of or maternal care for viable fetus in abdominal pregnancy (O833, O367) |
| Known or suspected disproportion of maternal and/or fetal origin | SMR02 | Delivery episode of pregnancy/birth in question has an ICD-10 code for maternal care for known or suspected disproportion (O33) or an ICD-9 code for disproportion in pregnancy labour and delivery (653) |
| Tumour of corpus uteri | SMR02 | Delivery episode of pregnancy/birth in question has an ICD-10 code for maternal care for tumour of corpus uteri (O341) or an ICD-9 code for tumors of body of uterus complicating pregnancy childbirth or the puerperium (6541) |
| Pre-labor non-elective cesarean section | SMR02 | Pregnancy/birth in question has code for non-elective caesarean section in mode of delivery field (8) AND duration of labour field=0 |
| Antepartum stillbirth | NRS stillbirths | Pregnancy/birth in question has code for antepartum stillbirth in period of death field (1) |
| **Exposures** |  |  |
| Elective repeat cesarean section (ERCS) | SMR02 | Pregnancy/birth in question has code for elective caesarean section in mode of delivery field (7) and number of previous caesarean sections ≥1 according to criteria for identifying ≥1 previous caesarean sections |
| Planned vaginal birth after previous cesarean section (planned VBAC) | SMR02 | Pregnancy/birth in question has code for vaginal delivery in mode of delivery field (0, 1, 2, 3, 4, A, B, C, D or E) OR code for non-elective caesarean section in mode of delivery field (8) AND duration of labour field ≥1 hour and number of previous caesarean sections ≥1 according to criteria for identifying ≥1 previous caesarean sections. Duration of labour defined as the length of time the state of labour lasts from its onset to the delivery of the placenta, expressed as the number of completed hours. |
| Planned VBAC without labor induction | SMR02 | Criteria for Planned VBAC AND code for none in induction of labour field (0) of pregnancy/birth in question |
| Planned VBAC with labor induction | SMR02 | Criteria for Planned VBAC AND code for induction of labour using artificial rupture of membranes (ARM), oxytocics, ARM & oxytocics, prostaglandins, prostaglandins & ARM, prostaglandins & oxytocics, prostaglandins & ARM & oxytocics or other method in induction of labour field (1-8) of pregnancy/birth in question. |
| Vaginal birth after previous cesarean section (VBAC) | SMR02 | Pregnancy/birth in question has code for vaginal delivery in mode of delivery field (0, 1, 2, 3, 4, A, B, C, D or E) and number of previous caesarean sections ≥1 according to criteria for identifying ≥1 previous caesarean sections |
| In-labor non-elective repeat cesarean section | SMR02 | Pregnancy/birth in question has code for non-elective caesarean section in mode of delivery field (8) AND duration of labour field ≥1 hour and number of previous caesarean sections ≥1 according to criteria for identifying ≥1 previous caesarean sections |
| **Outcomes** | | |
| Surgery for pelvic organ prolapse | SMR01 | \| **OPCS-4 code** \| **Description** \| **Paired with** \| \| --- \| --- \| --- \| \| P132 \| Female perineorrhaphy \| ICD-10 or ICD-9 code for genital prolapse (N81, N993, 618) as the main diagnosis \| \| P133 \| Female perineoplasty \| \| P134 \| Closure of fistula of female perineum \| \| P135 \| Female perineotomy NEC \| \| P171 \| Total colpectomy \| \| P172 \| Partial colpectomy \| \| P181 \| Complete colpocleisis \| \| P182 \| Partial colpocleisis \| \| P188 \| Other specified other obliteration of vagina \| \| P189 \| Unspecified other obliteration of vagina \| \| P213 \| Vaginoplasty NEC \| \| P214 \| Vaginoplasty in presence of uterus for absent vagina \| \| P215 \| Vaginoplasty using olive \| \| P22 \| Repair of prolapse of vagina and amputation of cervix uteri \| - \| \| P23 \| Other repair of prolapse of vagina \| - \| \| P24 \| Repair of vault of vagina \| - \| \| P288 \| Other specified repair of prolapse of vagina \| - \| \| P289 \| Unspecified repair of prolapse of vagina \| - \| \| P308 \| Other specified other repair of vault of vagina \| - \| \| P309 \| Unspecified other repair of vault of vagina \| - \| \| Q011 \| Amputation of cervix uteri \| ICD-10 or ICD-9 code for genital prolapse (N81, N993, 618) as the main diagnosis \| \| Q018 \| Other specified excision of cervix uteri \| \| Q071 \| Abdominal hysterocolpectomy and excision of periuterine tissue \| \| Q072 \| Abdominal hysterectomy and excision of periuterine tissue NEC \| \| Q073 \| Abdominal hysterocolpectomy NEC \| \| Q074 \| Total abdominal hysterectomy NEC \| \| Q075 \| Subtotal abdominal hysterectomy \| \| Q078 \| Other specified abdominal excision of uterus \| \| Q079 \| Unspecified abdominal excision of uterus \| \| Q081 \| Vaginal hysterocolpectomy and excision of periuterine tissue \| \| Q082 \| Vaginal hysterectomy and excision of periuterine tissue NEC \| \| Q083 \| Vaginal hysterocolpectomy NEC \| \| Q088 \| Other specified vaginal excision of uterus \| \| Q089 \| Unspecified vaginal excision of uterus \| - \| \| Q204 \| Vaginofixation of uterus \| - \| \| Q541 \| Suspension of uterus NEC \| - \| \| Q544 \| Suspension of uterus using mesh NEC \| - \| \| Q545 \| Sacrohysteropexy \| - \| \| Q546 \| Infracoccygeal hysteropexy \| - \|  \| **OPCS-3 code** \| **Description** \| **Paired with** \| \| --- \| --- \| --- \| \| 691 \| Extended hysterectomy \| ICD-10 or ICD-9 code for genital prolapse (N81, N993, 618) as the main diagnosis \| \| 692 \| Radical hysterectomy \| \| 6921 \| Radical hysterectomy: with pelvic lymphadenectomy \| \| 693 \| Vaginal hysterectomy \| \| 6931 \| Vaginal hysterectomy: total \| \| 6932 \| Vaginal hysterectomy: radical \| \| 6933 \| Vaginal hysterectomy: with repair of prolapse \| **-** \| \| 694 \| Sub-total hysterectomy \| ICD-10 or ICD-9 code for genital prolapse (N81, N993, 618) as the main diagnosis \| \| 696 \| Hysterectomy, not elsewhere classified \| \| 6961 \| Hysterectomy, not elsewhere classified: total hysterectomy \| \| 702 \| Hysteropexy \| - \| \| 710 \| Colporrhaphy: with repair of pelvic organs \| - \| \| 711 \| Colporrhaphy: with repair of pelvic floor \| - \| \| 712 \| Colporrhaphy: with amputation of cervix \| - \| \| 713 \| Colporrhaphy, not elsewhere classified \| - \| \| 714 \| Reconstruction and repair of vagina \| ICD-10 or ICD-9 code for genital prolapse (N81, N993, 618) as the main diagnosis \| \| 722 \| Obliteration of vagina \| \| 7221 \| Obliteration of vagina: obliteration of cul-de-sac \| \| 7222 \| Obliteration of vagina: colpoclesis, total obliteration \| \| 775 \| 775 Repair of vagina and pelvic floor \| - \| |
| Surgery for urinary incontinence | SMR01 | \| **OPCS-4 code** \| **Description** \| **Paired with** \| \| --- \| --- \| --- \| \| A70 \| Neurostimulation of peripheral nerve \| ICD-10 OR ICD-9 code for stress incontinence or other urinary incontinence (N393, N394, 6256, 7883) as the main diagnosis \| \| M191 \| Construction of ileal conduit \| \| M192 \| Creation of urinary diversion to intestine NEC \| \| M193 \| Revision of urinary diversion \| \| M194 \| Cutaneous ureterostomy NEC \| \| M195 \| Revision of ureterostomy stoma \| \| M198 \| Other specified urinary diversion \| \| M199 \| Unspecified urinary diversion \| \| M241 \| Construction of continent catheterisable intestinal pouch NEC \| \| M242 \| Construction of continent catheterisable intestinal pouch with continent cystostomy NEC \| \| M243 \| Construction of continent catheterisable intestinal pouch with continent cystostomy using appendix \| \| M244 \| Construction of continent catheterisable intestinal pouch with continent cystostomy using ileum \| \| M245 \| Creation of continent cystostomy NEC \| \| M246 \| Creation of continent cystostomy using appendix \| \| M247 \| Creation of continent cystostomy using ileum \| \| M248 \| Other specified other urinary diversion \| \| M344 \| Simple cystectomy \| \| M348 \| Other specified total excision of bladder \| \| M349 \| Unspecified total excision of bladder \| \| M35 \| Partial excision of bladder \| \| M36 \| Enlargement of bladder \| \| M371 \| Cystourethroplasty \| ICD-10 OR ICD-9 code for stress incontinence, other urinary incontinence or fistulae involving female genital tract (N393, N394, N82, 6256, 7883, 619) as the main diagnosis \| \| M372 \| Repair of vesicocolic fistula \| - \| \| M375 \| Repair of fistula of bladder NEC \| - \| \| M378 \| Other specified other repair of bladder \| ICD-10 OR ICD-9 code for stress incontinence, other urinary incontinence or fistulae involving female genital tract (N393, N394, N82, 6256, 7883, 619) as the main diagnosis \| \| M379 \| Unspecified other repair of bladder \| \| M416 \| Detrusor myotomy \| ICD-10 OR ICD-9 code for stress incontinence or other urinary incontinence (N393, N394, 6256, 7883) as the main diagnosis \| \| M43 \| Endoscopic operations to increase capacity of bladder \| \| M494 \| Introduction of therapeutic substance into bladder \| \| M495 \| Injection of therapeutic substance into bladder wall \| - \| \| M511 \| Abdominoperineal suspension of urethra \| - \| \| M512 \| Endoscopic suspension of neck of bladder \| - \| \| M518 \| Other specified combined abdominal and vaginal operations to support outlet of female bladder \| - \| \| M519 \| Unspecified combined abdominal and vaginal operations to support outlet of female bladder \| - \| \| M521 \| Suprapubic sling operation \| - \| \| M522 \| Retropubic suspension of neck of bladder \| - \| \| M523 \| Colposuspension of neck of bladder \| - \| \| M528 \| Other specified abdominal operations to support outlet of female bladder \| - \| \| M529 \| Unspecified abdominal operations to support outlet of female bladder \| - \| \| M531 \| Vaginal buttressing of urethra \| - \| \| M533 \| Introduction of tension-free vaginal tape \| - \| \| M536 \| Introduction of transobturator tape \| - \| \| M538 \| Other specified vaginal operations to support outlet of female bladder \| - \| \| M539 \| Unspecified vaginal operations to support outlet of female bladder \| - \| \| M542 \| Reconstruction of neck of female bladder NEC \| - \| \| M548 \| Other specified open operations on outlet of female bladder \| - \| \| M549 \| Unspecified open operations on outlet of female bladder \| - \| \| M552 \| Implantation of artificial urinary sphincter into outlet of female bladder \| - \| \| M553 \| Insertion of prosthetic collar around outlet of female bladder \| - \| \| M554 \| Maintenance of prosthetic collar around outlet of female bladder \| - \| \| M556 \| Insertion of retropubic device for female stress urinary incontinence NEC \| - \| \| M563 \| Endoscopic injection of inert substance into outlet of female bladder \| - \| \| M571 \| Introduction of vaginal tape NEC \| - \| \| M578 \| Other specified other vaginal operations to support outlet of female bladder \| - \| \| M579 \| Unspecified other vaginal operations to support outlet of female bladder \| - \| \| P251 \| Repair of vesicovaginal fistula \| - \| \| P252 \| Repair of urethrovaginal fistula \| - \| \| Y021 \| Implantation of prosthesis into organ NOC \| ICD-10 OR ICD-9 code for stress incontinence or other urinary incontinence (N393, N394, 6256, 7883) as the main diagnosis \| \| Y281 \| Insertion of synthetic mesh into organ NOC \| \| Y282 \| Insertion of biological mesh into organ NOC \| \| Y283 \| Insertion of composite mesh into organ NOC \|  \| **OPCS-3 code** \| **Description** \| **Paired with** \| \| --- \| --- \| --- \| \| 592 \| Urinary diversion: to bowel \| ICD-10 OR ICD-9 code for stress incontinence or other urinary incontinence (N393, N394, 6256, 7883) as the main diagnosis \| \| 5921 \| Urinary diversion: to bowel: uretero-ileostomy \| \| 5922 \| Urinary diversion: to bowel: uretero-colostomy \| \| 5923 \| Urinary diversion: to bowel: uretero-sigmoid proctostomy \| \| 5924 \| Urinary diversion: to bowel: uretero-sigmoidostomy with colostomy \| \| 593 \| Urinary diversion: anastomosis, not elsewhere classified \| \| 594 \| Urinary diversion: other, not elsewhere classified \| \| 5941 \| Urinary diversion: other, not elsewhere classified: secondary operation, not otherwise stated \| \| 5942 \| Urinary diversion: other, not elsewhere classified: repair of stomal opening \| \| 603 \| Resection of bladder neck \| \| 6031 \| Resection of bladder neck: open operation \| \| 6032 \| Resection of bladder neck: per-urethral \| \| 605 \| Repair or replacement of bladder \| \| 6051 \| Repair or replacement of bladder: augmentation of bladder \| \| 6052 \| Repair or replacement of bladder: bladder neck repair \| \| 6054 \| Reconstruction of bladder \| \| 618 \| Vesicourethral suspension \| - \| \| 6181 \| Vesicourethral suspension: urethrovesical plication \| - \| \| 6182 \| Vesicourethral suspension: putococcygeoplasty \| - \| \| 6183 \| Vesicourethral suspension: suprapubic sling \| - \| \| 6184 \| Vesicourethral suspension: retropubic suspension \| - \| \| 6185 \| Vesicourethral suspension: periurethral suspension \| - \| \| 6191 \| Implantation of electrical stimulator \| ICD-10 OR ICD-9 code for stress incontinence or other urinary incontinence (N393, N394, 6256, 7883) as the main diagnosis \| \| 6242 \| Repair of urethra: sling operation \| - \| \| 715 \| Repair of vaginal fistula \| - \| \| 7151 \| Repair of vaginal fistula: with reconstruction of urethra \| - \| |
|  |  |  |
| Surgery for rectal prolapse or fecal incontinence | SMR01 | \| **OPCS-4 code** \| **Description** \| **Paired with** \| \| --- \| --- \| --- \| \| H04 \| Total excision of colon and rectum \| ICD-10 or ICD-9 code for anal prolapse, rectal prolapse, faecal incontinence or nonorganic encopresis (K622, K623, R15, F981, 5691, 7876) \| \| H05 \| Total excision of colon \| \| H09 \| Excision of left hemicolon \| \| H10 \| Excision of sigmoid colon \| \| H192 \| Fixation of colon \| \| H29 \| Subtotal excision of colon \| \| H33 \| Excision of rectum \| \| H351 \| Anterior fixation of rectum \| \| H352 \| Posterior fixation of rectum using prosthetic material \| \| H353 \| Posterior fixation of rectum NEC \| \| H354 \| Fixation of rectum using fascia lata \| \| H355 \| Anterior fixation of rectum using prosthetic material \| - \| \| H358 \| Other specified fixation of rectum for prolapse \| - \| \| H359 \| Unspecified fixation of rectum for prolapse \| - \| \| H361 \| Abdominal repair of levator ani muscles \| - \| \| H368 \| Other specified other abdominal operations for prolapse of rectum \| - \| \| H369 \| Unspecified other abdominal operations for prolapse of rectum \| - \| \| H411 \| Rectosigmoidectomy and peranal anastomosis \| ICD-10 or ICD-9 code for anal prolapse, rectal prolapse, faecal incontinence or nonorganic encopresis (K622, K623, R15, F981, 5691, 7876) \| \| H412 \| Peranal excision of lesion of rectum \| \| H414 \| Peranal mucosal proctectomy and endoanal anastomosis \| \| H415 \| Peranal resection of rectum using staples \| - \| \| H421 \| Insertion of encircling suture around perianal sphincter \| - \| \| H422 \| Perineal plication of levator ani muscles and anal sphincters \| - \| \| H423 \| Insertion of supralevator sling \| - \| \| H425 \| Excision of mucosal prolapse of rectum NEC \| - \| \| H426 \| Perineal repair of prolapse of rectum NEC \| - \| \| H427 \| Rubber band ligation of prolapsed rectal mucosa \| - \| \| H428 \| Other specified perineal operations for prolapse of rectum \| - \| \| H429 \| Unspecified perineal operations for prolapse of rectum \| - \| \| H501 \| Posterior repair of anal sphincter \| - \| \| H502 \| Anterior repair of anal sphincter \| - \| \| H508 \| Other specified repair of anus \| ICD-10 or ICD-9 code for anal prolapse, rectal prolapse, faecal incontinence or nonorganic encopresis (K622, K623, R15, F981, 5691, 7876) \| \| H509 \| Unspecified repair of anus \| \| H551 \| Laying open of low anal fistula \| - \| \| H552 \| Laying open of high anal fistula \| - \| \| H553 \| Laying open of anal fistula NEC \| - \| \| H554 \| Insertion of seton into high anal fistula and partial laying open of track HFQ \| - \| \| H555 \| Fistulography of anal fistula \| - \| \| H556 \| Probing of perineal fistula \| - \| \| H557 \| Repair of anal fistula using plug \| - \| \| H558 \| Other specified other operations on perianal region \| ICD-10 or ICD-9 code for anal prolapse, rectal prolapse, faecal incontinence or nonorganic encopresis (K622, K623, R15, F981, 5691, 7876) \| \| H559 \| Unspecified other operations on perianal region \| \| H568 \| Other specified other operations on anus \| \| H569 \| Unspecified other operations on anus \| \| H57 \| Other operations on the anal sphincter to control continence \| - \| \| P253 \| Repair of rectovaginal fistula \| - \|  \| **OPCS-3 code** \| **Description** \| **Paired with** \| \| --- \| --- \| --- \| \| 460 \| Colectomy and resection, not elsewhere classified \| ICD-10 or ICD-9 code for anal prolapse, rectal prolapse, faecal incontinence or nonorganic encopresis (K622, K623, R15, F981, 5691, 7876) \| \| 4601 \| Colectomy and resection, not elsewhere classified: with enterostomy \| \| 4602 \| Colectomy and resection, not elsewhere classified: with anastomosis \| \| 4605 \| Colectomy and resection, not elsewhere classified: excision colostomy mucosa \| \| 461 \| Complete colectomy \| \| 462 \| Colostomy, primary \| \| 4621 \| Colostomy, primary: exteriorization \| \| 4625 \| Colostomy, primary: petineal \| \| 4691 \| Other operations on intestine, not elsewhere classified: fixation \| \| 471 \| Excision of rectum, not elsewhere classified \| \| 4711 \| Excision of rectum, not elsewhere classified: abdominal-anal operation or anastomosis \| \| 4712 \| Excision of rectum, not elsewhere classified: intra pelvic proctectomy \| \| 4713 \| Excision of rectum, not elsewhere classified: sphincter preservation \| \| 4714 \| Excision of rectum, not elsewhere classified: pull through resection \| \| 472 \| Abdomino-perineal excision of rectum \| \| 474 \| Repair and anastomosis of rectum \| \| 4742 \| Repair and anastomosis of rectum: rectocele \| \| 4743 \| Repair and anastomosis of rectum: closure of stoma \| \| 4744 \| Repair and anastomosis of rectum: proctoplasty \| \| 475 \| Repair of rectal prolapse \| - \| \| 481 \| Incision of other anal lesion \| ICD-10 or ICD-9 code for anal prolapse, rectal prolapse, faecal incontinence or nonorganic encopresis (K622, K623, R15, F981, 5691, 7876) \| \| 4811 \| Incision of other anal lesion: incision of fistula \| \| 4812 \| Incision of other anal lesion: division of anal sphincter \| \| 482 \| Excision of anal fistula \| - \| \| 486 \| Repair of anus \| ICD-10 or ICD-9 code for anal prolapse, rectal prolapse, faecal incontinence or nonorganic encopresis (K622, K623, R15, F981, 5691, 7876) \| \| 4861 \| Repair of anus: repair of sphincter \| - \| \| 4862 \| Repair of anus: sphincterectomy \| ICD-10 or ICD-9 code for anal prolapse, rectal prolapse, faecal incontinence or nonorganic encopresis (K622, K623, R15, F981, 5691, 7876) \| \| 776 \| Repair of perineum and sphincter ani \| - \| |
| Any pelvic floor surgery | SMR01 | Any of codes for pelvic organ prolapse, urinary incontinence, rectal prolapse or fecal incontinence surgery as specified above. |
| **Socio-demographic characteristics** | |  |
| Maternal age at birth (years) | SMR02 | Derived from mother’s *date of birth* and *date of delivery* of pregnancy/birth in question  *(Treated as a continuous variable in analysis but grouped into following categories for descriptive purposes: <25, 25-29, 30-34, 35-39, ≥40)* |
| Mother’s country of birth | NRS live and stillbirths | *Mother’s Country of Birth* field recorded at the time birth in question was registered  *(Categorized as: UK, Non-UK)* |
| Marital status/registration type | NRS live and stillbirths | *Parents married indicator* field recorded at the time birth in question was registered  *(Categorized as: Married, Joint registration, Sole registration)* |
| Area deprivation | NRS live and stillbirths | Postcode of residence recorded at the time birth in question was registered was used to derive Carstairs scores – a measure of area deprivation derived from Census data, available for the years 1981, 1991, 2001 and 2011[6]. Births from 1981-1985 used the 1981 Carstairs scores and births from 1986-1996 used the 1991 Carstairs scores. These scores were converted into quintiles based on the total population of Scotland at each time period, with 1 representing the least deprived and 5 the most deprived. |
| **Maternal medical and obstetric-related characteristics** | | |
| Previous mode(s) of birth | SMR02 | Derived from parity (number of previous pregnancies resulting in either a live birth or stillbirth) and number of previous caesarean sections in pregnancy/birth in question. Mode of birth in all of a woman’s previous delivery records was also examined.  *(Main analysis categorized as a binary variable: Cesarean section(s) only, Cesarean section(s) and vaginal birth(s); Sensitivity analysis using only the subgroup of women who have all their previous births in the SMR02 categorized as: Cesarean section(s) only, all pre-labor, Cesarean section(s) only, at least on in-labor, Cesarean section(s) and spontaneous vaginal birth(s), Cesarean section(s) and at least one assisted (instrumental) vaginal birth)* |
| Parity | SMR02 | Parity field (number of previous pregnancies resulting in either a live birth or stillbirth) in pregnancy/birth in question. The parity field was cross-checked with the number of previous delivery records observed to date. Where the parity value found to be less than the number of previous delivery records observed to date, it was overwritten with the higher number  *(Treated as a continuous variable in main analysis but grouped into following categories for descriptive purposes: 1, ≥2)* |
| Inter-pregnancy interval (months) | SMR02 | Derived from interval between *date of delivery* of pregnancy/birth in question and *date of delivery* of previous delivery minus gestational age at birth in question  *(Treated as a continuous variable in analysis but grouped into following categories for descriptive purposes: <12, 12-23, ≥24)* |
| Pre-existing or gestational diabetes mellitus | SMR02 | Antenatal or delivery episodes of pregnancy/birth in question has a code for pre-existing or gestational diabetes mellitus in *diabetes* field (1-3) OR an ICD-10 or ICD-9 code for pre-existing or gestational diabetes mellitus (O24, E10-E11, 6480, 250)  *(Categorized as: No, Yes)* |
| Birth weight (grams) | SMR02 | Birthweight field. Implausible birth weights identified as those birth weights for gestational age that were more than twice the interquartile range below and above the first and third quartile, respectively, using the sex-specific birth weight for gestational age centiles reported by Bonellie et at[7]. Implausible values were set to missing.  *(Main analysis: birth weight of child in* pregnancy/birth in question, *treated as a continuous variable in analysis but grouped into following categories for descriptive purposes: <2,500, 2500-3999, ≥4000; Sensitivity analysis using only the subgroup of women who have all their previous births in the SMR02: birth weight of child in pregnancy/birth in question or in any previous pregnancy/birth ≥4000 categorized as No, Yes)* |
| Any previous multiple birth(s)  *(Only included in sensitivity analysis conducted using the subgroup of women who have all their previous births in the SMR02)* | NRS live births and stillbirths or SMR02 | Identified from numbirths field OR from number of births in this pregnancy field if numbirths field missing, using all of a woman’s previous delivery records  *(Categorized as: No, Yes)* |
| Any previous third- or fourth- degree perineal tear(s)  *(Only included in sensitivity analysis conducted using the subgroup of women who have all their previous births in the SMR02)* |  | Delivery episodes of any previous deliveries have a code for third- or fourth-degree tear in *tears* field (3 or 4) OR ICD-10 or ICD-9 code for third- or fourth-degree perineal laceration during delivery (O702-0703, 6642, 6643) OR OPCS-4 or OPCS-3 code for repair of obstetric laceration of perineum and sphincter of anus or repair of obstetric laceration of perineum and sphincter and mucosa of anus (R322, R325, 776).  *(Categorized as: No, Yes)* |
| Any previous shoulder dystocia  *(Only included in sensitivity analysis conducted using the subgroup of women who have all their previous births in the SMR02)* |  | Delivery episodes of any previous deliveries have an ICD-10 code for obstructed labour due to shoulder dystocia (O660) or an ICD-9 code for shoulder (girdle) dystocia (6604)  *(Categorized as: No, Yes)* |

Cesarean section/Elective repeat cesarean section

Vaginal birth/planned VBAC

Pelvic floor surgery

No pelvic floor surgery at time of censoring/end of follow-up period

Period of time in ERCS group

Period of time in planned VBAC group

CS/

ERCS

CS

ERCS

CS

ERCS

Woman 1

V/

VBAC

ERCS

VBAC

V

Woman 2

CS

Woman 3

VBAC

VBAC

VBAC

Woman 4

CS

Woman emigrated or died

Woman 5

CS

ERCS

VBAC

CS

Woman 6

VBAC

VBAC

Did not meet study eligibility criteria e.g. preterm birth

VBAC

ERCS

CS

Woman 7

Did not meet study eligibility criteria e.g. preterm birth

Calendar time

31 Dec 2016 (end of follow-up period)

**Fig A. Schematic to explain time-varying exposure model used, allowing inclusion of more than one birth per woman in the study cohort**

If a woman had >1 birth during the follow-up period, the time to event was restarted after each successive birth, with the exposures and covariates (e.g previous mode(s) of birth) treated as time-varying variables that were re-assessed at each pregnancy/birth. Abbreviations: CS, cesarean section; ERCS, elective repeat cesarean section; V, vaginal birth; VBAC, vaginal birth after previous cesarean

**Text A. Extract from application to the Public Benefit and Privacy Panel for Health and Social Care Scotland, taken from application submitted in March 2018**

*We did not publish or pre-register an analysis plan, but a summary of the proposed study exposures, outcomes and statistical methods was included as part of the application to the Public Benefit and Privacy Panel for Health and Social Care Scotland to obtain the data. An extract of relevant text from this application is shown below. Please note this was submitted as part of a larger programme of research that aimed to investigate the short and longer-term outcomes for women and their children according to planned mode of delivery after previous cesarean section. As such more data was requested than was included in this manuscript, and the below text reflects that of a data application as opposed to a formal pre-registered analysis plan.*

**Background**: Many countries, including the UK, have seen a rise in their caesarean section rate (now ~25% of all birhs in UK) leading to an increasing proportion of women embarking on a subsequent pregnancy with a history of previous caesarean section (estimated at ~100,000 women per year in UK). Broad policy consensus in high-income countries supports offering pregnant women who have had previous caesarean delivery a choice between planning to have another caesarean, known as an elective repeat caesarean section (ERCS), or attempting a vaginal delivery, known as a vaginal birth after previous caesarean (VBAC, also known as trial of labour). This is provided that they do not have contraindications to planned VBAC such as placenta praevia (low-lying placenta), where a caesarean section is clearly necessary. Current UK guidelines [8] advise that such women should be counselled about the risks and benefits of ERCS compared to planned VBAC to help them make informed decisions with respect to this aspect of their maternity care. Furthermore, previous research suggests that many women would find it helpful to have access to accurate, comprehensive and well-balanced information about the risks and benefits of the different ways of giving birth after a previous caesarean section when making what many view as a very difficult decision. However, a number of significant limitations have been highlighted with the existing evidence, and there have been calls for methodologically rigorous studies to assess both the short and longer-term outcomes for women and their children of intended mode of delivery after prior caesarean section and thus fill this evidence gap.

-Objective 1b, to investigate the effect of elective repeat caesarean section (ERCS) compared to planned vaginal birth after caesarean section (VBAC) on the longer-term health outcome for the mother

Pelvic floor

**Study design**: Retrospective population-based cohort study.

**Participants/Eligibility criteria**: Include all births from 1983-1996 (cohort selection period) to women who have had one or more previous caesarean sections, identified from the Scottish Morbidity Record Maternity Inpatient and Day Case dataset (SMR02). Dependent on the data available and the data quality, the research team plan to try and exclude women with contraindications to planned VBAC based on current UK guidelines [8] (e.g. placenta praevia in latest pregnancy in cohort selection period or uterine rupture in any previous pregnancy).

**Methods**: The following linked data sources will be used to derive information on exposures, outcomes, potential cofounding, mediating or moderating factors and take account of censoring if appropriate for births meeting the eligibility criteria: SMR02 records from 1981-2016 (births in cohort selection period to eligible women and all their pregnancy records occurring before and after cohort selection period); National Records of Scotland (NRS) deaths for mother from 1983-2016; Scottish Morbidity Record General/ Acute Inpatient and Day Case dataset (SMR01) for mother from 1981-2016; Community Health Index (CHI) database from 1983-2016; NRS live births and stillbirths from 1981-2016.

**Outcomes**: Surgical treatment for urinary incontinence, pelvic organ prolapse, rectal prolapse or faecal incontinence (data source: SMR01)

**Statistical analysis:** A descriptive analysis of the comparison groups, outcomes, potential confounders, mediators and moderators will first be conducted. Internal and external validation of the data will be performed by, for example, doing checks such as plausible birthweight for gestational age and comparing whether the prevalence of data items is consistent with published sources where available. Missing data and the factors associated with “missingness” will be assessed to determine the most appropriate method of addressing this in the analysis (e.g. complete case analysis, multiple imputation, inverse probability weighting). Standard statistical methods will be used to estimate the effect of **intended mode of delivery** on outcomes: logistic regression will be used to estimate odds ratios for rare binary outcomes (e.g. peripartum hysterectomy); modified Poisson regression will be used to estimate risk ratios for more common binary outcomes (e.g. breastfeeding); or Cox regression will be used to allow for follow-up time and take account of censoring if appropriate using information on deaths and emigration ascertained from the National Records of Scotland Deaths data and the CHI database respectively. In a series of secondary analyses, for comparative purpose, outcomes will also be examined according to **actual mode of delivery**. The influence of potential confounders, mediators and moderators on the association between intended mode of delivery and outcomes will be explored.

The main analyses will be confined to singleton births of cephalic presentation at term (≥37 weeks gestation). This is the main group of women current UK guidelines [8] recommend are candidates for and should be counselled about intended mode of delivery following previous caesarean section.

**Table C. Complete case analysis of outcomes following planned VBAC compared to ERCS**

| **Outcomes** | **Unadjusted model HR (95% CI)** | **Base model^1^ HR (95% CI)** | **Model A^2^ HR (95% CI)** | **Model B^3^ HR (95% CI)** |
| --- | --- | --- | --- | --- |
| Any pelvic floor surgery | **2.70 (2.32-3.14)** | **2.70 (2.32-3.14)** | **2.79 (2.40-3.25)** | **2.36 (1.97-2.81)** |
|  | **P<0.001** | **P<0.001** | **P<0.001** | **P<0.001** |
| Surgery for pelvic organ prolapse | **3.80 (3.01-4.81)** | **3.80 (3.00-4.80)** | **3.95 (3.12-4.99)** | **2.88 (2.19-3.78)** |
|  | **P<0.001** | **P<0.001** | **P<0.001** | **P<0.001** |
| Surgery for urinary incontinence | **2.51 (2.02-3.12)** | **2.51 (2.02-3.12)** | **2.60 (2.09-3.24)** | **2.34 (1.81-3.02)** |
|  | **P<0.001** | **P<0.001** | **P<0.001** | **P<0.001** |
| Surgery for rectal prolapse or fecal incontinence | 1.18 (0.82-1.70) | 1.18 (0.82-1.71) | 1.18 (0.81-1.71) | 1.10 (0.71-1.71) |
|  | P=0.382 | P=0.369 | P=0.380 | P=0.658 |

^1^ Base model adjusted for year of birth only.

^2^ Model A adjusted for year of birth & socio-demographic factors (maternal age, mother's country of birth, marital status, & area deprivation).

^3^ Model B adjusted for variables in Model A & additionally adjusted for maternal medical & obstetric-related factors (previous mode(s) of birth – categorized as cesarean section only/ cesarean section and vaginal birth(s), parity, inter-pregnancy interval, diabetes, birth weight of child in pregnancy/birth in question treated as a continuous variable).

Bold text indicates statistically significant findings at the 5% level.

Abbreviations: CI, confidence interval; ERCS, elective repeat cesarean section; HR, Hazard ratio; VBAC, vaginal birth after previous cesarean

**Table D. Complete case analysis of outcomes following planned VBAC with and without labor induction compared to ERCS**

| **Outcomes** | **Planned VBAC without labor induction vs. ERCS** | | | |  | | **Planned VBAC with labor induction vs. ERCS** | | | |
| --- | --- | --- | --- | --- | --- | --- | --- | --- | --- | --- |
|  | **Unadjusted model HR (95% CI)** | **Base model^1^ HR (95% CI)** | **Model A^2^ HR (95% CI)** | **Model B^3^ HR (95% CI)** | |  | **Unadjusted model HR (95% CI)** | **Base model^1^ HR (95% CI)** | **Model A^2^ HR (95% CI)** | **Model B^3^ HR (95% CI)** |
| Any pelvic floor surgery | **2.65** | **2.65** | **2.74** | **2.31** | |  | **2.86** | **2.85** | **2.93** | **2.52** |
|  | **(2.27-3.10)** | **(2.27-3.10)** | **(2.35-3.21)** | **(1.92-2.77)** | |  | **(2.37-3.44)** | **(2.37-3.44)** | **(2.43-3.54)** | **(2.03-3.12)** |
|  | **P<0.001** | **P<0.001** | **P<0.001** | **P<0.001** | |  | **P<0.001** | **P<0.001** | **P<0.001** | **P<0.001** |
| Surgery for pelvic organ prolapse | **3.74** | **3.74** | **3.89** | **2.80** | |  | **4.00** | **3.99** | **4.12** | **3.13** |
|  | **(2.95-4.75)** | **(2.94-4.75)** | **(3.06-4.95)** | **(2.12-3.70)** | |  | **(3.04-5.28)** | **(3.02-5.26)** | **(3.12-5.44)** | **(2.28-4.30)** |
|  | **P<0.001** | **P<0.001** | **P<0.001** | **P<0.001** | |  | **P<0.001** | **P<0.001** | **P<0.001** | **P<0.001** |
| Surgery for urinary incontinence | **2.42** | **2.43** | **2.52** | **2.27** | |  | **2.78** | **2.78** | **2.86** | **2.57** |
|  | **(1.94-3.03)** | **(1.94-3.04)** | **(2.01-3.16)** | **(1.75-2.96)** | |  | **(2.12-3.64)** | **(2.12-3.64)** | **(2.18-3.75)** | **(1.88-3.50)** |
|  | **P<0.001** | **P<0.001** | **P<0.001** | **P<0.001** | |  | **P<0.001** | **P<0.001** | **P<0.001** | **P<0.001** |
| Surgery for rectal prolapse or fecal incontinence | 1.20 | 1.21 | 1.20 | 1.12 | |  | 1.10 | 1.11 | 1.11 | 1.03 |
|  | (0.82-1.77) | (0.82-1.77) | (0.81-1.78) | (0.72-1.77) | |  | (0.64-1.90) | (0.65-1.91) | (0.65-1.91) | (0.56-1.92) |
|  | P=0.350 | P=0.340 | P=0.352 | P=0.612 | |  | P=0.721 | P=0.700 | P=0.703 | P=0.916 |

^1^ Base model adjusted for year of birth only.

^2^ Model A adjusted for year of birth & socio-demographic factors (maternal age, mother's country of birth, marital status, & area deprivation).

^3^ Model B adjusted for variables in Model A & additionally adjusted for maternal medical & obstetric-related factors (previous mode(s) of birth – categorized as cesarean section only/ cesarean section and vaginal birth(s), parity, inter-pregnancy interval, diabetes, birth weight of child in pregnancy/birth in question treated as a continuous variable).

Bold text indicates statistically significant findings at the 5% level.

Abbreviations: CI, confidence interval; ERCS, elective repeat cesarean section; HR, Hazard ratio; VBAC, vaginal birth after previous cesarean

**Table E. Complete case analysis of outcomes according to actual mode of birth – planned VBAC and had a VBAC and planned VBAC but had in-labor non-elective repeat cesarean section compared to ERCS**

| **Outcomes** | **Planned VBAC and had VBAC vs. ERCS** | | | |  | | **Planned VBAC but had in-labor non-elective repeat cesarean section vs. ERCS** | | | |
| --- | --- | --- | --- | --- | --- | --- | --- | --- | --- | --- |
|  | **Unadjusted model HR (95% CI)** | **Base model^1^ HR (95% CI)** | **Model A^2^ HR (95% CI)** | **Model B^3^ HR (95% CI)** | |  | **Unadjusted model HR (95% CI)** | **Base model^1^ HR (95% CI)** | **Model A^2^ HR (95% CI)** | **Model B^3^ HR (95% CI)** |
| Any pelvic floor surgery | **3.01** | **3.01** | **3.12** | **2.64** | |  | 1.20 | 1.20 | 1.23 | 1.23 |
|  | **(2.59-3.51)** | **(2.59-3.50)** | **(2.67-3.63)** | **(2.20-3.16)** | |  | (0.92-1.57) | (0.92-1.57) | (0.94-1.61) | (0.91-1.67) |
|  | **P<0.001** | **P<0.001** | **P<0.001** | **P<0.001** | |  | P=0.176 | P=0.180 | P=0.123 | P=0.182 |
| Surgery for pelvic organ prolapse | **4.34** | **5.67** | **4.52** | **3.25** | |  | 1.21 | 1.11 | 1.25 | 1.34 |
|  | **(3.43-5.49)** | **(4.49-7.16)** | **(3.57-5.72)** | **(2.45-4.30)** | |  | (0.79-1.85) | (0.74-1.67) | (0.81-1.91) | (0.84-2.12) |
|  | **P<0.001** | **P<0.001** | **P<0.001** | **P<0.001** | |  | P=0.381 | P=0.62 | P=0.310 | P=0.219 |
| Surgery for urinary incontinence | **2.76** | **2.76** | **2.87** | **2.62** | |  | 1.30 | 1.30 | 1.34 | 1.26 |
|  | **(2.21-3.43)** | **(2.21-3.43)** | **(2.30-3.57)** | **(2.01-3.41)** | |  | (0.89-1.88) | (0.89-1.88) | (0.92-1.94) | (0.81-1.93) |
|  | **P<0.001** | **P<0.001** | **P<0.001** | **P<0.001** | |  | P=0.172 | P=0.172 | P=0.128 | P=0.303 |
| Surgery for rectal prolapse or fecal incontinence | 1.29 | 1.29 | 1.29 | 1.23 | |  | 0.64 | 0.65 | 0.64 | 0.57 |
|  | (0.89-1.87) | (0.89-1.88) | (0.89-1.89) | (0.79-1.93) | |  | (0.30-1.37) | (0.30-1.38) | (0.30-1.38) | (0.24-1.39) |
|  | P=0.185 | P=0.177 | P=0.183 | P=0.354 | |  | P=0.253 | P=0.259 | P=0.257 | P=0.219 |

^1^ Base model adjusted for year of birth only.

^2^ Model A adjusted for year of birth & socio-demographic factors (maternal age, mother's country of birth, marital status, & area deprivation).

^3^ Model B adjusted for variables in Model A & additionally adjusted for maternal medical & obstetric-related factors (previous mode(s) of birth – categorized as cesarean section only/ cesarean section and vaginal birth(s), parity, inter-pregnancy interval, diabetes, birth weight of child in pregnancy/birth in question treated as a continuous variable).

Bold text indicates statistically significant findings at the 5% level.

Abbreviations: CI, confidence interval; ERCS, elective repeat cesarean section; HR, Hazard ratio; VBAC, vaginal birth after previous cesarean

**Table F. E-values for the observed associations between planned mode of birth after previous cesarean section (planned VBAC vs. ERCS) and pelvic floor outcomes***

| **Outcome** | **E-value for HR point estimate^a^** | **E-value for CI limit of HR^b^** |
| --- | --- | --- |
| Any pelvic floor surgery | 4.19 | 3.48 |
|  |  |  |
| Surgery for pelvic organ prolapse | 5.79 | 4.38 |
|  |  |  |
| Surgery for urinary incontinence | 3.95 | 2.98 |

* The observed associations are the fully adjusted HRs shown in Table 2. See VanderWeele & Ding[9] for formula used to calculate E-values.

^a^ The E-values for the point estimates are the minimum strength of association on the HR scale that an unmeasured confounder would need to have with both the exposure & outcome, above & beyond the measured covariates, to fully explain the observed associations between planned mode of birth after previous cesarean section (planned VBAC vs. ERCS) & the pelvic floor outcomes.

^b^ The E-values for the limit of the 95% CI closest to the null covey the minimum strength of association on the HR scale that an unmeasured confounder would need to have with both the exposure & outcome, above & beyond the measured covariates, to move the CI to include the null value.

Abbreviations: CI, confidence interval; ERCS, elective repeat cesarean section; HR, Hazard ratio; VBAC, vaginal birth after previous cesarean

**Table G. E-values for the observed associations between planned mode of birth after previous cesarean section (planned VBAC with and without labor induction vs. ERCS) and pelvic floor outcomes***

| **Outcome** | **E-value for HR point estimate^a^** | **E-value for CI limit of HR^b^** |
| --- | --- | --- |
| **Planned VBAC with labor induction** |  |  |
| Any pelvic floor surgery | 4.33 | 3.46 |
|  |  |  |
| Surgery for pelvic organ prolapse | 5.87 | 4.21 |
|  |  |  |
| Surgery for urinary incontinence | 4.33 | 3.10 |
| **Planned VBAC without labor induction** |  |  |
| Any pelvic floor surgery | 4.15 | 3.41 |
|  |  |  |
| Surgery for pelvic organ prolapse | 5.77 | 4.33 |
|  |  |  |
| Surgery for urinary incontinence | 3.80 | 2.85 |

* The observed associations are the fully adjusted HRs shown in Table 3. See VanderWeele & Ding[9] for formula used to calculate E-values.

^a^ The E-values for the point estimates are the minimum strength of association on the HR scale that an unmeasured confounder would need to have with both the exposure & outcome, above & beyond the measured covariates, to fully explain the observed associations between planned mode of birth after previous cesarean section (planned VBAC vs. ERCS) & the pelvic floor outcomes.

^b^ The E-values for the limit of the 95% CI closest to the null covey the minimum strength of association on the HR scale that an unmeasured confounder would need to have with both the exposure & outcome, above & beyond the measured covariates, to move the CI to include the null value.

Abbreviations: CI, confidence interval; ERCS, elective repeat cesarean section; HR, Hazard ratio; VBAC, vaginal birth after previous cesarean

**Table H. E-values for the observed associations between actual mode of birth after previous cesarean section (planned VBAC and had a VBAC and planned VBAC but had in-labor non-elective repeat cesarean section compared vs. ERCS) and pelvic floor outcomes***

| **Outcome** | **E-value for HR point estimate^a^** | **E-value for CI limit of HR^b^** |
| --- | --- | --- |
| **Planned VBAC and had VBAC** |  |  |
| Any pelvic floor surgery | 4.82 | 3.99 |
|  |  |  |
| Surgery for pelvic organ prolapse | 6.82 | 5.13 |
|  |  |  |
| Surgery for urinary incontinence | 4.46 | 3.37 |

* The observed associations are the fully adjusted HRs shown in Table 4. See VanderWeele & Ding[9] for formula used to calculate E-values.

^a^ The E-values for the point estimates are the minimum strength of association on the HR scale that an unmeasured confounder would need to have with both the exposure & outcome, above & beyond the measured covariates, to fully explain the observed associations between planned mode of birth after previous cesarean section (planned VBAC vs. ERCS) & the pelvic floor outcomes.

^b^ The E-values for the limit of the 95% CI closest to the null covey the minimum strength of association on the HR scale that an unmeasured confounder would need to have with both the exposure & outcome, above & beyond the measured covariates, to move the CI to include the null value.

Abbreviations: CI, confidence interval; ERCS, elective repeat cesarean section; HR, Hazard ratio; VBAC, vaginal birth after previous cesarean

**Table I. Outcomes following planned VBAC compared to ERCS in women who had all their previous births in the SMR02**

| **Outcomes** | **ERCS  Number of events/person-years (Rate per 100 person-years)** | **Planned VBAC Number of events/person-years (Rate per 100 person-years)** | **Unadjusted model HR (95% CI)** | **Base model^1^ HR (95% CI)** | **Model A^2^ HR (95% CI)** | **Model B^3^ HR (95% CI)** |
| --- | --- | --- | --- | --- | --- | --- |
| Any pelvic floor surgery | 129/211,861 | 572/375,083 | **2.55 (2.10-3.08)** | **2.55 (2.11-3.09)** | **2.62 (2.16-3.17)** | **2.36 (1.92-2.91)** |
|  | (0.61) | (1.53) | **P<0.001** | **P<0.001** | **P<0.001** | **P<0.001** |
| Surgery for pelvic organ prolapse | 53/212,631 | 287/378,371 | **3.08 (2.30-4.13)** | **3.09 (2.30-4.15)** | **3.18 (2.37-4.28)** | **2.87 (2.09-3.96)** |
|  | (0.25) | (0.76) | **P<0.001** | **P<0.001** | **P<0.001** | **P<0.001** |
| Surgery for urinary incontinence | 56/212,817 | 283/378,305 | **2.90 (2.18-3.87)** | **2.92 (2.19-3.89)** | **3.03 (2.27-4.03)** | **2.73 (2.01-3.70)** |
|  | (0.26) | (0.75) | **P<0.001** | **P<0.001** | **P<0.001** | **P<0.001** |
| Surgery for rectal prolapse or fecal incontinence | 30/213,016 | 58/380,842 | 1.10 (0.71-1.70) | 1.11 (0.71-1.72) | 1.11 (0.71-1.73) | 0.96 (0.61-1.52) |
|  | (0.14) | (0.15) | P=0.686 | P=0.647 | P=0.659 | P=0.865 |

^1^ Base model adjusted for year of birth only.

^2^ Model A adjusted for year of birth & socio-demographic factors (maternal age, mother's country of birth, marital status, & area deprivation).

^3^ Model B adjusted for variables in Model A & additionally adjusted for maternal medical & obstetric-related factors (previous mode(s) of birth – categorized as: cesarean section(s) only, all pre-labor/ cesarean section(s) only, at least on in-labor/ cesarean section(s) and spontaneous vaginal birth(s)/ cesarean section(s) and at least one assisted (instrumental) vaginal birth, parity, inter-pregnancy interval, diabetes, birth weight of child in pregnancy/birth in question or any previous pregnancy/birth ≥4000g, any previous multiple birth(s), any previous third- or fourth-degree perineal tear(s), any previous shoulder dystocia).

Bold text indicates statistically significant findings at the 5% level.

Abbreviations: CI, confidence interval; ERCS, elective repeat cesarean section; HR, Hazard ratio; VBAC, vaginal birth after previous cesarean

| **Outcomes** | **ERCS** | **Planned VBAC without labor induction** | | | | | | |  | **Planned VBAC with labor induction** | | | | |
| --- | --- | --- | --- | --- | --- | --- | --- | --- | --- | --- | --- | --- | --- | --- |
|  | **Number of events/ person-years (Rate per 100 person-years)** | **Number of events/ person-years (Rate per 100 person-years)** | **Unadjusted model HR (95% CI)** | **Base model^1^ HR (95% CI)** | **Model A^2^ HR (95% CI)** | **Model B^3^ HR (95% CI)** |  | **Number of events/ person-years (Rate per 100 person-years)** | | | **Unadjusted model HR (95% CI)** | **Base model^1^ HR (95% CI)** | **Model A^2^ HR (95% CI)** | **Model B^3^ HR (95% CI)** |
| Any pelvic floor surgery | 129/211,861 | 421/291,470 | **2.41** | **2.42** | **2.48** | **2.25** |  | 151/83,603 | | | **3.02** | **3.03** | **3.10** | **2.74** |
|  | (0.61) | (1.44) | **(1.98-2.94)** | **(1.98-2.95)** | **(2.03-3.03)** | **(1.82-2.79)** |  | (1.81) | | | **(2.39-3.83)** | **(2.40-3.84)** | **(2.45-3.92)** | **(2.14-3.51)** |
|  |  |  | **P<0.001** | **P<0.001** | **P<0.001** | **P<0.001** |  |  | | | **P<0.001** | **P<0.001** | **P<0.001** | **P<0.001** |
| Surgery for pelvic organ prolapse | 53/212,631 | 206/293,879 | **2.84** | **2.85** | **2.94** | **2.68** |  | 81/84,482 | | | **3.90** | **3.91** | **4.02** | **3.49** |
|  | (0.25) | (0.70) | **(2.10-3.85)** | **(2.10-3.86)** | **(2.17-3.99)** | **(1.93-3.72)** |  | (0.96) | | | **(2.76-5.52)** | **(2.76-5.54)** | **(2.84-5.70)** | **(2.42-5.04)** |
|  |  |  | **P<0.001** | **P<0.001** | **P<0.001** | **P<0.001** |  |  | | | **P<0.001** | **P<0.001** | **P<0.001** | **P<0.001** |
| Surgery for urinary incontinence | 56/212,817 | 205/293,858 | **2.71** | **2.72** | **2.82** | **2.55** |  | 78/84,437 | | | **3.60** | **3.61** | **3.72** | **3.35** |
|  | (0.26) | (0.70) | **(2.01-3.64)** | **(2.02-3.66)** | **(2.10-3.80)** | **(1.87-3.50)** |  | (0.92) | | | **(2.55-5.07)** | **(2.56-5.09)** | **(2.64-5.24)** | **(2.35-4.78)** |
|  |  |  | **P<0.001** | **P<0.001** | **P<0.001** | **P<0.001** |  |  | | | **P<0.001** | **P<0.001** | **P<0.001** | **P<0.001** |
| Surgery for rectal prolapse or fecal incontinence | 30/213,016 | 46/295,557 | 1.12 | 1.13 | 1.13 | 0.99 |  | 12/85,276 | | | 1.01 | 1.02 | 1.02 | 0.88 |
|  | (0.14) | (0.16) | (0.71-1.77) | (0.72-1.80) | (0.71-1.80) | (0.61-1.58) |  | (0.14) | | | (0.52-1.98) | (0.52-2.00) | (0.52-2.01) | (0.44-1.77) |
|  |  |  | P=0.632 | P=0.594 | P=0.609 | P=0.951 |  |  | | | P=0.970 | P=0.944 | P=0.944 | P=0.717 |

**Table J. Outcomes following planned VBAC with and without labor induction compared to ERCS in women who had all their previous births in the SMR02**

^1^ Base model adjusted for year of birth only.

^2^ Model A adjusted for year of birth & socio-demographic factors (maternal age, mother's country of birth, marital status, & area deprivation).

^3^ Model B adjusted for variables in Model A & additionally adjusted for maternal medical & obstetric-related factors (previous mode(s) of birth – categorized as: cesarean section(s) only, all pre-labor/ cesarean section(s) only, at least on in-labor/ cesarean section(s) and spontaneous vaginal birth(s)/ cesarean section(s) and at least one assisted (instrumental) vaginal birth, parity, inter-pregnancy interval, diabetes, birth weight of child in pregnancy/birth in question or any previous pregnancy/birth ≥4000g, any previous multiple birth(s), any previous third- or fourth-degree perineal tear(s), any previous shoulder dystocia).

Bold text indicates statistically significant findings at the 5% level.

Abbreviations: CI, confidence interval; ERCS, elective repeat cesarean section; HR, Hazard ratio; VBAC, vaginal birth after previous cesarean

| **Outcomes** | **ERCS** | **Planned VBAC and had VBAC** | | | | | | |  | **Planned VBAC but had in-labor non-elective repeat cesarean section** | | | | |
| --- | --- | --- | --- | --- | --- | --- | --- | --- | --- | --- | --- | --- | --- | --- |
|  | **Number of events/ person-years (Rate per 100 person-years)** | **Number of events/ person-years (Rate per 100 person-years)** | **Unadjusted model HR (95% CI)** | **Base model^1^ HR (95% CI)** | **Model A^2^ HR (95% CI)** | **Model B^3^ HR (95% CI)** |  | **Number of events/ person-years (Rate per 100 person-years)** | | | **Unadjusted model HR (95% CI)** | **Base model^1^ HR (95% CI)** | **Model A^2^ HR (95% CI)** | **Model B^3^ HR (95% CI)** |
| Any pelvic floor surgery | 129/211,861 | 527/307,326 | **2.87** | **2.88** | **2.96** | **2.71** |  | 45/67,756 | | | 1.10 | 1.10 | 1.12 | 1.14 |
|  | (0.61) | (1.71) | **(2.37-3.48)** | **(2.37-3.49)** | **(2.44-3.60)** | **(2.19-3.35)** |  | (0.66) | | | (0.78-1.54) | (0.79-1.55) | (0.79-1.57) | (0.81-1.61) |
|  |  |  | **P<0.001** | **P<0.001** | **P<0.001** | **P<0.001** |  |  | | | P=0.584 | P=0.569 | P=0.526 | P=0.448 |
| Surgery for pelvic organ prolapse | 53/212,631 | 270/310,288 | **3.54** | **3.55** | **3.68** | **3.38** |  | 17/68,083 | | | 1.01 | 1.01 | 1.02 | 1.09 |
|  | (0.25) | (0.87) | **(2.63-4.75)** | **(2.64-4.77)** | **(2.73-4.96)** | **(2.43-4.70)** |  | (0.25) | | | (0.58-1.74) | (0.58-1.75) | (0.59-1.77) | (0.63-1.91) |
|  |  |  | **P<0.001** | **P<0.001** | **P<0.001** | **P<0.001** |  |  | | | P=0.982 | P=0.973 | P=0.930 | P=0.749 |
| Surgery for urinary incontinence | 56/212,817 | 260/310,272 | **3.26** | **3.27** | **3.42** | **3.13** |  | 23/68,034 | | | 1.30 | 1.31 | 1.33 | 1.36 |
|  | (0.26) | (0.84) | **(2.44-4.35)** | **(2.45-4.37)** | **(2.56-4.56)** | **(2.29-4.27)** |  | (0.34) | | | (0.80-2.11) | (0.81-2.13) | (0.82-2.16) | (0.83-2.22) |
|  |  |  | **P<0.001** | **P<0.001** | **P<0.001** | **P<0.001** |  |  | | | P=0.287 | P=0.276 | P=0.251 | P=0.217 |
| Surgery for rectal prolapse or fecal incontinence | 30/213,016 | 53/312,645 | 1.22 | 1.24 | 1.23 | 1.09 |  | 5/68,197 | | | 0.52 | 0.53 | 0.53 | 0.48 |
|  | (0.14) | (0.17) | (0.78-1.91) | (0.79-1.94) | (0.78-1.94) | (0.68-1.75) |  | (0.07) | | | (0.20-1.35) | (0.21-1.37) | (0.21-1.37) | (0.19-1.24) |
|  |  |  | P=0.382 | P=0.356 | P=0.367 | P=0.716 |  |  | | | P=0.181 | P=0.190 | P=0.191 | P=0.128 |

**Table K. Outcomes according to actual mode of birth – planned VBAC and had a VBAC and planned VBAC but had in-labor non-elective repeat cesarean section compared to ERCS in women who had all their previous births in the SMR02**

^1^ Base model adjusted for year of birth only.

^2^ Model A adjusted for year of birth & socio-demographic factors (maternal age, mother's country of birth, marital status, & area deprivation).

^3^ Model B adjusted for variables in Model A & additionally adjusted for maternal medical & obstetric-related factors (previous mode(s) of birth – categorized as: cesarean section(s) only, all pre-labor/ cesarean section(s) only, at least on in-labor/ cesarean section(s) and spontaneous vaginal birth(s)/ cesarean section(s) and at least one assisted (instrumental) vaginal birth, parity, inter-pregnancy interval, diabetes, birth weight of child in pregnancy/birth in question or any previous pregnancy/birth ≥4000g, any previous multiple birth(s), any previous third- or fourth-degree perineal tear(s), any previous shoulder dystocia).

Bold text indicates statistically significant findings at the 5% level.

Abbreviations: CI, confidence interval; ERCS, elective repeat cesarean section; HR, Hazard ratio; VBAC, vaginal birth after previous cesarean

**Table L. Rate and hazard ratio of any pelvic floor surgery by the exposures of interest in the subgroup of women who gave birth between 1^st^ January 1983 and 31^st^ December 1986**

|  | **Number of events/person-years (Rate per 100 person-years)** | **Unadjusted model HR (95% CI)** | **Base model^1^ HR (95% CI)** | **Model A^2^ HR (95% CI)** | **Model B^3^ HR (95% CI)** |
| --- | --- | --- | --- | --- | --- |
| **Planned VBAC vs. ERCS** |  |  |  |  |  |
| ERCS | 74/90,348 (0.82) | 1 | 1 | 1 | 1 |
| Planned VBAC | 307/170,617 (1.80) | **2.26 (1.75-2.91) P<0.001** | **2.22 (1.73-2.86) P<0.001** | **2.27 (1.76-2.93) P<0.001** | **1.92 (1.47-2.51) P<0.001** |
| **Planned VBAC with and without labor induction vs. ERCS** |  |  |  |  |  |
| ERCS | 74/90,348 (0.82) | 1 | 1 | 1 | 1 |
| Planned VBAC without labor induction | 229/126,561 (1.81) | **2.28 (1.75-2.96) P<0.001** | **2.24 (1.72-2.91) P<0.001** | **2.29 (1.76-2.97) P<0.001** | **1.05 (1.48-2.56) P<0.001** |
| Planned VBAC with labor induction | 78/44,053 (1.77) | **2.21 (1.61-3.03) P<0.001** | **2.18 (1.59-3.00) P<0.001** | **2.22 (1.62-3.06) P<0.001** | **1.85 (1.33-2.57) P<0.001** |
| **Planned VBAC and had a VBAC and planned VBAC but had in-labor non-elective repeat cesarean section vs. ERCS** |  |  |  |  |  |
| ERCS | 74/90,348 (0.82) | 1 | 1 | 1 | 1 |
| Planned VBAC and had VBAC | 286/139,884 (2.04) | **2.58 (1.99-3.33) P<0.001** | **2.54 (1.97-3.28) P<0.001** | **2.59 (2.01-3.35) P<0.001** | **2.21 (1.69-2.91) P<0.001** |
| Planned VBAC but had in-labor non-elective repeat cesarean section | 21/30,733 (0.68) | 0.84 (0.52-1.37) P=0.493 | 0.84 (0.52-1.37) P=0.495 | 0.87 (0.53-1.41) P=0.573 | 0.86 (0.53-1.41) P=0.548 |

^1^ Base model adjusted for year of birth only.

^2^ Model A adjusted for year of birth & socio-demographic factors (maternal age, mother's country of birth, marital status, & area deprivation).

^3^ Model B adjusted for variables in Model A & additionally adjusted for maternal medical & obstetric-related factors (previous mode(s) of birth – categorized as cesarean section only/ cesarean section and vaginal birth(s), parity, inter-pregnancy interval, diabetes, birth weight of child in pregnancy/birth in question treated as a continuous variable).

Bold text indicates statistically significant findings at the 5% level.

Abbreviations: CI, confidence interval; ERCS, elective repeat cesarean section; HR, Hazard ratio; VBAC, vaginal birth after previous cesarean

**Table M. Outcomes following planned VBAC compared to ERCS, defining planned VBAC as birth vaginally or by non-electve cesarean section with a duration of labor of >4 hours (rather than ≥1 hour)**

| **Outcomes** | **ERCS  Number of events/person-years (Rate per 100 person-years)** | **Planned VBAC Number of events/person-years (Rate per 100 person-years)** | **Unadjusted model HR (95% CI)** | **Base model^1^ HR (95% CI)** | **Model A^2^ HR (95% CI)** | **Model B^3^ HR (95% CI)** |
| --- | --- | --- | --- | --- | --- | --- |
| Any pelvic floor surgery | 206/313,960 (0.66) | 935/525,056 (1.78) | **2.76 (2.37-3.21)** | **2.75 (2.37-3.20)** | **2.84 (2.44-3.30)** | **2.44 (2.07-2.87)** |
|  |  |  | **P<0.001** | **P<0.001** | **P<0.001** | **P<0.001** |
| Surgery for pelvic organ prolapse | 81/315,369 (0.26) | 525/530,096 (0.99) | **3.90 (3.09-4.94)** | **3.90 (3.08-4.92)** | **4.06 (3.21-5.14)** | **3.28 (2.55-4.23)** |
|  |  |  | **P<0.001** | **P<0.001** | **P<0.001** | **P<0.001** |
| Surgery for urinary incontinence | 100/315,399 (0.32) | 419/530,988 (0.79) | **2.54 (2.04-3.15)** | **2.54 (2.04-3.15)** | **2.61 (2.10-3.25)** | **2.29 (1.81-2.89)** |
|  |  |  | **P<0.001** | **P<0.001** | **P<0.001** | **P<0.001** |
| Surgery for rectal prolapse or fecal incontinence | 42/316,011 (0.13) | 85/535,000 (0.16) | 1.21 (0.84-1.75) | 1.22 (0.84-1.76) | 1.22 (0.84-1.77) | 1.16 (0.76-1.75) |
|  |  |  | P=0.309 | P=0.295 | P=0.300 | P=0.491 |

^1^ Base model adjusted for year of birth only.

^2^ Model A adjusted for year of birth & socio-demographic factors (maternal age, mother's country of birth, marital status, & area deprivation).

^3^ Model B adjusted for variables in Model A & additionally adjusted for maternal medical & obstetric-related factors (previous mode(s) of birth – categorized as cesarean section only/cesarean section and vaginal birth(s), parity, inter-pregnancy interval, diabetes, birth weight of child in pregnancy/birth in question treated as a continuous variable).

Bold text indicates statistically significant findings at the 5% level.

Abbreviations: CI, confidence interval; ERCS, elective repeat cesarean section; HR, Hazard ratio; VBAC, vaginal birth after previous cesarean

**References**

1. National Records of Scotland. Quality of Data Obtained from the Registration of Births, Stillbirths, Marriages, Civil Partnerships and Deaths [Accessed 16 May 2018]. Available from: <https://www.nrscotland.gov.uk/files//statistics/vital-events/quality-data-obtained-from-registration-of-ve.pdf> [Accessed 16 May 2018].

2. Information Services Division Scotland. Births in Scottish Hospitals Technical Report Publication date - 28 November 2017. Edinburgh,: 2017.

3. Information Services Division Scotland. Data Quality Assurance Assessment of Maternity Data (SMR02) 2008-2009 Edinburgh,: 2010.

4. Information Services Division Scotland. Assessment of SMR02 (Maternity Inpatient and Day Case) Data Scotland 2017-2018. Edinburgh: 2019.

5. Information Services Division Scotland. Data Quality Assurance Assessment of SMR01 Data Scotland 2014-2015. Edinburgh: 2016.

6. Public Health Scotland. Deprivation guidance for analysts, version 3.4. 2020.

7. Bonellie S, Chalmers J, Gray R, Greer I, Jarvis S, Williams C. Centile charts for birthweight for gestational age for Scottish singleton births. BMC Pregnancy Childbirth. 2008;8:5. Epub 2008/02/27. doi: 10.1186/1471-2393-8-5. PubMed PMID: 18298810; PubMed Central PMCID: PMCPMC2268653.

8. Royal College of Obstetricians and Gynaecologists. Birth After Previous Caesarean Birth, Green-top Guideline No. 45 London, 2015.

9. VanderWeele TJ, Ding P. Sensitivity Analysis in Observational Research: Introducing the E-Value. Annals of Internal Medicine. 2017;167(4):268-74. Epub 2017/07/12. doi: 10.7326/M16-2607. PubMed PMID: 28693043.
